# Fine-scale grassland land-cover classification using UAV RGB and multispectral imagery with staged feature selection

**DOI:** 10.3389/fpls.2026.1956870

**Published:** 2026-10-06

**Authors:** Ying Liu, Mingyang Jiang, Haiyan Zhao, Mingyang Sun, Baoyou Qiao, Qiang Guan, Wen Du

**Affiliations:** 1 College of Computer Science and Technology, Inner Mongolia Minzu University, Tongliao, Inner Mongolia, China; 2 College of Information and Electrical Engineering, Shenyang Agricultural University, Shenyang, Liaoning, China

**Keywords:** feature selection, grassland, land-cover classification, multispectral imagery, UAV

## Abstract

**Introduction:**

Accurate mapping of grassland vegetation and bare land is important for grassland degradation assessment and ecological management. This study investigated the effectiveness of integrating visible and multispectral imagery with staged feature selection for fine-scale grassland land-cover classification.

**Methods:**

Four representative grassland land-cover classes, including Caragana microphylla, Artemisia scoparia, Agropyron cristatum, and bare land, were selected as classification categories. Spectral bands, vegetation indices, and texture metrics were combined to construct a multi-source feature pool, and different feature optimization strategies and classification models were systematically compared.

**Results:**

Multi-source feature fusion improved the discrimination of spectrally similar land-cover classes, while staged feature selection provided a workflow for reducing cross-source feature redundancy while retaining complementary information. The model with the highest observed validation accuracy selected only ten features from the original feature pool and achieved an overall accuracy of 91.54% with a Kappa coefficient of 88.72%. Compared with direct feature fusion, the optimized feature subset substantially reduced feature dimensionality while maintaining comparable classification accuracy. The resulting classification maps showed broadly consistent spatial patterns, with differences mainly reflected in local boundary and fragmentation patterns.

**Discussion:**

Under the conditions evaluated in this study, these findings indicate that staged feature selection provides a compact and interpretable feature optimization strategy for fine-scale UAV-based grassland land-cover classification. However, the data were acquired from a single study area during a single growing season, and the robustness of the framework across different regions, years, and phenological stages remains to be validated.

## 1 Introduction

Grassland ecosystems are among the most important terrestrial ecosystems worldwide, covering approximately 40% of the Earth’s land surface. They perform critical ecological functions, including carbon sequestration, soil and water conservation, and biodiversity maintenance, and constitute an indispensable component of the terrestrial ecosystem service supply system (Petermann and Buzhdygan, 2021; Li et al., 2025). However, under the long-term disturbance of human activities such as overgrazing and unsustainable land-use practices, grassland ecosystems have experienced a series of degradation problems, including vegetation community deterioration, intensified soil erosion, soil nutrient depletion, and substantial biodiversity loss (Lin et al., 2022; Jiang et al., 2024; Li et al., 2025). These issues not only threaten regional ecological security but also constrain the sustainable socioeconomic development of pastoral areas. Vegetation composition and dominant species succession are important indicators for characterizing grassland degradation processes and assessing degradation levels. Accurate monitoring of vegetation composition and fine-scale grassland vegetation classification are therefore essential for grassland degradation assessment, resource management, and ecological restoration, and carry considerable practical significance (Dong et al., 2022; Ali and Kaul, 2025).

In recent years, unmanned aerial vehicle (UAV)-based low-altitude remote sensing technology has been widely applied in ecological environmental monitoring and precision agriculture owing to its advantages of high spatial resolution, strong operational flexibility, and relatively low cost (Jay et al., 2017; Fernández-Guisuraga et al., 2022). UAV platforms equipped with visible-light and multispectral sensors have become important tools for vegetation classification studies. However, due to the limited spectral information provided by sensors, different grassland vegetation types often exhibit highly similar spectral responses in certain wavelength bands. Moreover, in complex grassland environments, the co-occurrence of multiple vegetation species and variations in illumination conditions can further affect classification accuracy. Consequently, relying solely on a single type of feature is often insufficient for accurate vegetation identification (Wu et al., 2024; Meng et al., 2025), highlighting the need for multidimensional information fusion approaches to improve classification performance.

In grassland remote sensing studies, multi-source feature fusion, such as the integration of spectral bands, vegetation indices, and texture information, has become an important approach for mitigating the effects of spectral mixing and habitat heterogeneity while improving classification accuracy (Xu et al., 2019; Zhou et al., 2023). Compared with individual features, multidimensional features can enhance the representation of land-cover characteristics from multiple aspects, including spectral responses, spatial structures, and vegetation physiological conditions (Kong et al., 2024). However, the direct stacking of a large number of cross-source features inevitably introduces high correlation and multicollinearity. Such information redundancy not only increases the risk of the “curse of dimensionality” and substantially raises computational costs, but also weakens the generalization capability of classification models (Ma and Ju, 2022). Therefore, designing effective feature fusion and dimensionality reduction strategies to identify optimal feature subsets with high discriminative power and low redundancy has become a key challenge in high-accuracy grassland vegetation classification. Nevertheless, the sequence in which feature fusion and selection are performed may substantially affect how redundant information is retained and complementary information is utilized. Their combined impact on classification performance remains insufficiently understood.

At present, multi-source feature optimization mainly follows two paradigms: fusion-before-selection and selection-before-fusion. However, both strategies exhibit inherent limitations when dealing with complex land-cover classification tasks (Wang et al., 2015; Zheng et al., 2021a). Under the fusion-before-selection strategy, traditional filter-based feature selection methods typically rely on the statistical significance of individual features while largely neglecting interactions among features. As a result, features with weak individual discriminative power but strong complementary effects in combination may be mistakenly discarded, making it difficult to effectively eliminate highly correlated redundant information across different data sources. In contrast, the selection-before-fusion strategy reduces the search space by independently filtering different feature groups. Nevertheless, such isolated processing may hinder the exploitation of synergistic information between multispectral and visible-light features at deeper feature levels. Therefore, systematic comparisons are needed to clarify the effects of different dimensionality-reduction and feature optimization strategies on multi-source feature classification.

Although various advanced feature selection algorithms have been introduced for high-dimensional remote sensing data analysis (Cheng et al., 2010; Jiang et al., 2022), significant overlap in spectral and structural characteristics often exists among different grassland vegetation categories (e.g., shrubs and herbaceous vegetation) in UAV-based fine-scale grassland vegetation classification. Few studies have systematically compared different feature selection strategies with respect to their abilities to suppress redundancy, uncover hidden complementary information, and improve classification performance. Therefore, it is necessary to comprehensively evaluate multiple feature selection methods with distinct selection mechanisms under a unified experimental framework, to clarify the relationships among feature fusion strategies, feature selection algorithms, and classifier adaptability.

To address these gaps, this study constructed a 46-dimensional multi-source feature pool by integrating spectral bands, vegetation indices, and texture metrics from UAV RGB and multispectral imagery. We systematically compared two feature optimization workflows, namely fusion-before-selection (one-stage) and selection-before-fusion (two-stage), using three established feature selection algorithms (SPA, mRMR, and ReliefF) and three classifiers (SVM, KNN, and LR) for fine-scale classification of four typical grassland land-cover types. The methodological contribution of this study lies in the workflow design and systematic evaluation of source-wise selection before cross-source refinement, rather than in the development of a new feature-selection algorithm. The main contributions of this study are as follows. First, we provide a comparative evaluation of how fusion order affects feature redundancy reduction, complementary information retention, and classification performance in UAV RGB–multispectral feature fusion. Second, we show that the effectiveness of a fusion strategy depends on the feature selection algorithm, indicating that two-stage selection is not universally superior but can be advantageous when paired with an appropriate selection method. Third, from an application perspective, we identify a compact and effective feature subset for distinguishing shrub, herbaceous vegetation, and bare land in a typical grassland, thereby supporting fine-scale grassland vegetation mapping and degradation-related monitoring.

## 2 Materials and methods

### 2.1 Study area

The study area is located in Manghatu Gacha, Gerichaolu Sumu, Zhalute Banner, Tongliao City, Inner Mongolia, China (approximately 44° 37’ N, 120° 45’ E; **Figure 1**), at an elevation of approximately 482 m. The region has a temperate semi-arid continental climate, with a mean annual temperature of approximately 5.5°C, annual precipitation of 300–400 mm, and annual evaporation exceeding 1800 mm. Nearly 70% of precipitation occurs from June to August; annual sunshine duration is approximately 3000 h, and the frost-free period is 115–130 d.

**Figure 1:**
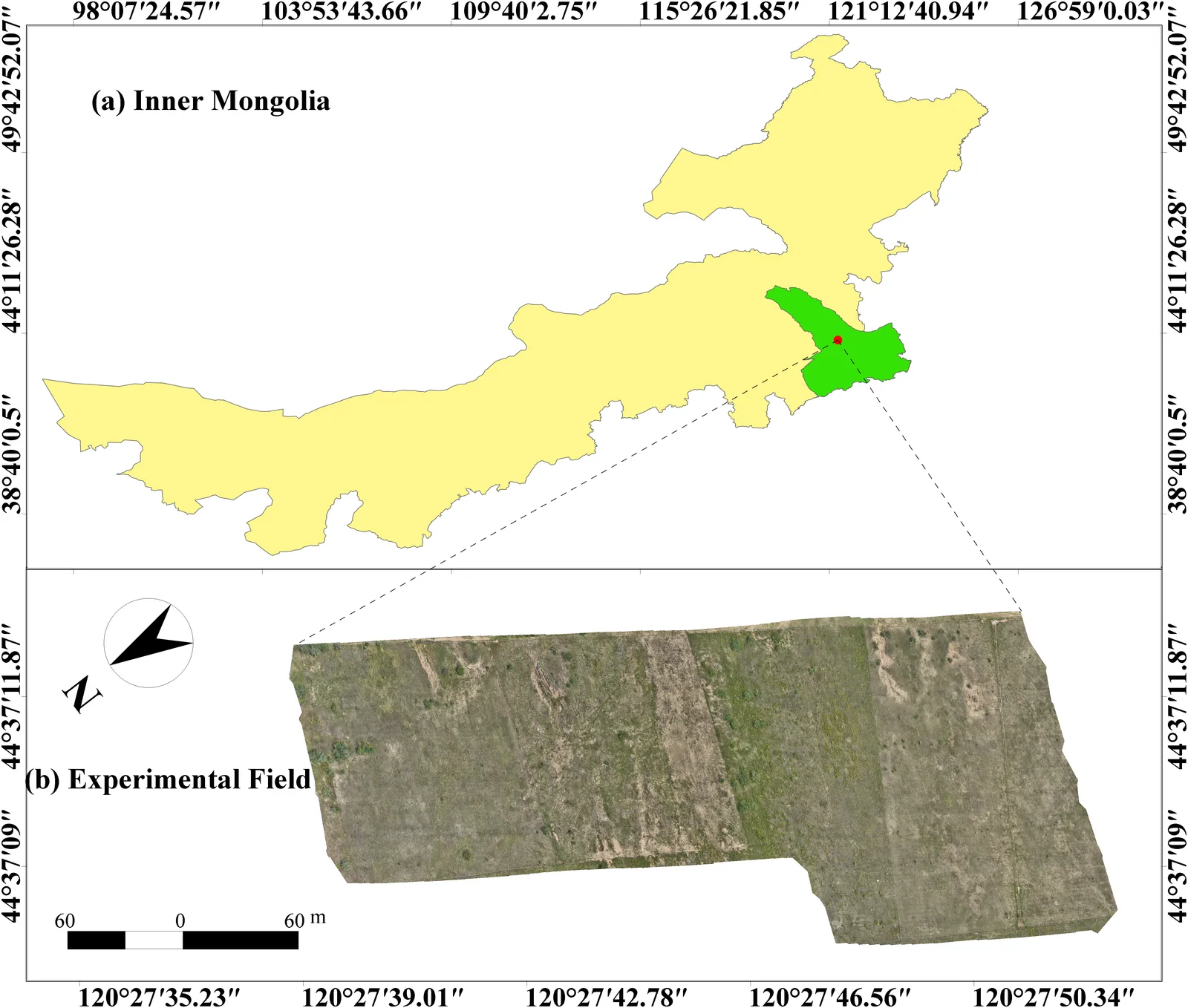
Location of the study area. **(a)** Location of the study site in Inner Mongolia; **(b)** Drone imagery of the experimental field.

The study area is a temperate mountain grassland dominated by sandy loam (pH approximately 7.6). Agropyron cristatum is the principal species, accompanied by Lespedeza davurica, Cleistogenes squarrosa, and Carex duriuscula. Field surveys identified Caragana microphylla, Agropyron cristatum, Artemisia scoparia, and bare land as the four target classes. Their heterogeneous, patchy distribution provides a suitable setting for evaluating fine-scale land-cover classification.

### 2.2 Data acquisition

#### 2.2.1 UAV platform

The DJI Mavic 3M UAV was employed as the remote sensing platform for data acquisition, enabling the simultaneous collection of high-resolution RGB and multispectral imagery. The UAV is equipped with a 20-megapixel RGB camera featuring a 4/3 CMOS sensor and a four-band multispectral camera. The multispectral sensor includes four spectral bands: green (560 ± 16 nm), red (650 ± 16 nm), red-edge (730 ± 16 nm), and near-infrared (860 ± 26 nm). Each multispectral image contains 2592 × 1944 pixels. In addition, the UAV is equipped with a Global Navigation Satellite System (GNSS) and a real-time kinematic (RTK) module, providing centimeter-level positioning accuracy.

#### 2.2.2 Image acquisition

Image acquisition was conducted between 11:00 and 12:00 (Beijing Time) on 19 August 2025. The flight altitude was set to 30 m, with both forward and side overlap ratios maintained at 80%. Weather conditions during data collection were clear with low wind speed, and flight routes were planned to ensure complete coverage of the study area. During image acquisition, the onboard spectral sunlight sensor of the DJI Mavic 3M recorded band-specific solar irradiance information in the image metadata. During multispectral reconstruction, DJI Terra used the sunlight-sensor records together with image metadata and manufacturer-provided calibration parameters to compensate for illumination variation and generate the four-band multispectral orthomosaic. The multispectral bands, vegetation indices, and texture features used in this study were derived from the resulting irradiance-compensated multispectral products. RGB images were orthorectified and mosaicked separately, and RGB features and indices were calculated from the digital RGB values (0–255) of the RGB orthomosaic.

#### 2.2.3 Data preprocessing

After the UAV survey, the raw images were processed in DJI Terra software for aerial triangulation and orthorectification, generating RGB and multispectral orthomosaics of the study area. The RGB orthomosaic had a spatial resolution of approximately 0.87 cm/pixel, whereas the multispectral orthomosaic had a spatial resolution of approximately 1.54 cm/pixel. The generated orthomosaics exhibited minimal geometric distortion and clear texture details, meeting the requirements for subsequent fine-scale land-cover identification and feature extraction.

#### 2.2.4 Sample construction

Based on field survey data and visual interpretation of the high-resolution imagery, candidate areas corresponding to four land-cover classes—Caragana microphylla, Agropyron cristatum, Artemisia scoparia, and bare land—were identified in ENVI 5.6. Within the eligible areas of each class, ROI units were randomly sampled while maintaining approximately balanced class sizes. The dataset comprised 3,353 separate ROI units rather than pixels or sub-samples repeatedly extracted from a smaller number of ROIs, including 834 Caragana microphylla, 841 Agropyron cristatum, 842 Artemisia scoparia, and 836 bare-land ROIs. The extracted ROI patches ranged from 24 × 22 pixels to 130 × 120 pixels. ROI ground areas were not separately recorded in the original sampling records; therefore, the available image-patch dimensions are reported. Each complete ROI was assigned exclusively to either the training subset or the validation subset, and no ROI was divided between the two subsets. The data were allocated at an approximate ratio of 3:1. A *post hoc* spatial check showed a minimum boundary distance of 0 m between some training and validation ROIs. Because no predefined minimum separation distance or geographically blocked allocation rule was imposed during the original sampling, the resulting split should be regarded as an ROI-level holdout rather than a fully spatially independent validation design. Detailed sample statistics are presented in **Table 1**.

**Table 1:** Numbers of samples for different land-cover classes in the training and validation datasets.

Class	Training samples	Validation samples
Caragana microphylla	639	195
Artemisia scoparia	642	200
Agropyron cristatum	641	200
Bare land	639	197

### 2.3 Methods

#### 2.3.1 Overview of the methodology

The proposed workflow comprised four stages: data preparation, multi-source feature construction and selection, classification modeling, and accuracy assessment and mapping (**Figure 2**).

**Figure 2:**
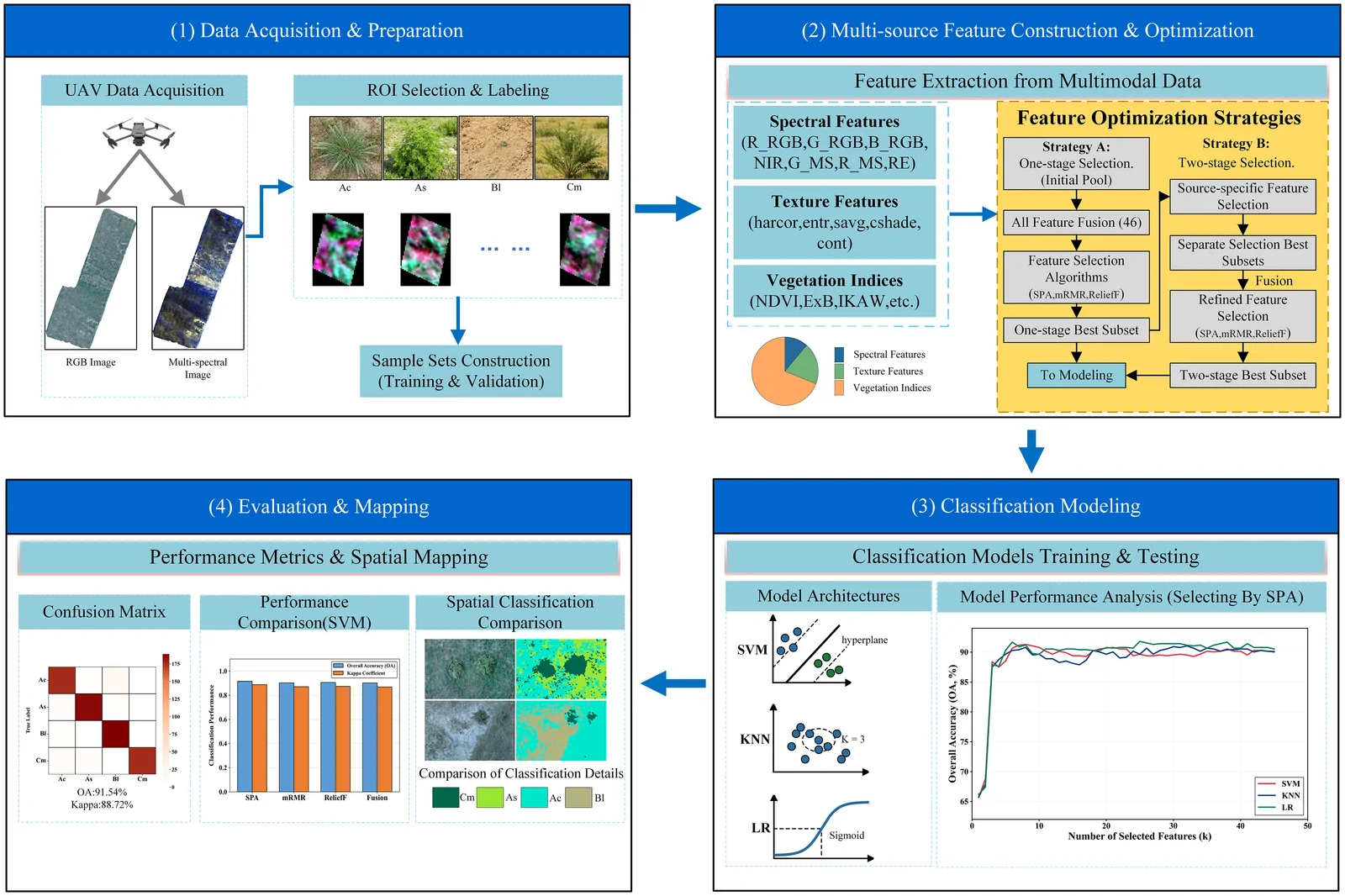
Workflow of the proposed classification framework.

1. Data acquisition and preparation: High-resolution RGB and multispectral imagery were acquired using a UAV platform. Combined with field survey data, regions of interest (ROIs) corresponding to Agropyron cristatum (Ac), Artemisia scoparia (As), bare land (Bl), and Caragana microphylla (Cm) were delineated in ENVI software to construct standardized training and validation datasets.
2. Multi-source feature construction and workflow comparison: First, spectral features, texture features, and derived vegetation indices were extracted from the RGB and multispectral imagery to establish an initial 46-dimensional feature pool. Subsequently, two feature optimization workflows were compared. Strategy A (one-stage strategy: fusion-before-selection) directly fused all extracted features and then identified the optimal feature subset. Strategy B (two-stage strategy: selection-before-fusion) first performed independent preliminary feature screening within each data source, followed by secondary refinement on the fused feature set to determine the final feature subset.
3. Classification modeling: The optimal feature subsets obtained from different optimization strategies were separately input into three classifiers, namely Support Vector Machine (SVM), K-Nearest Neighbors (KNN), and Logistic Regression (LR), for model training and testing. The dynamic relationships among feature optimization strategies, feature dimensionality, and classification performance were systematically analyzed.
4. Evaluation and mapping: Based on the independent validation dataset, confusion matrices, overall accuracy (OA), Kappa coefficient, and class-level metrics, including producer’s accuracy (PA), user’s accuracy (UA), and F1-score, were employed to quantitatively evaluate and compare the performance of different classification schemes. High-accuracy spatial classification maps were subsequently generated for local-scale visual validation and detailed spatial analysis.

#### 2.3.2 Feature construction

The feature set constructed in this study consisted of spectral features, texture features, and vegetation index features (**Table 2**). Spectral features included three visible-light bands from the RGB imagery (R_RGB, G_RGB, and B_RGB) and four bands from the multispectral imagery (NIR, G_MS, R_MS, and RE). A total of 29 vegetation indices were calculated, including 14 derived from RGB imagery and 15 derived from multispectral imagery.

**Table 2:** Extracted features used in this study.

Category	Features
Spectral features	R_RGB, G_RGB, B_RGB, NIR, G_MS, R_MS, RE.
Texture features	Haralick correlation (harcor), Entropy (entr), Sum average (savg),
	Cluster shade (cshade), Contrast (cont).
Vegetation Indices	ExG (Liu et al., 2025), ExB (Liu et al., 2025), ExR (Liu et al., 2025), MExG(Wang and Zhang, 2020), VDVI (Xu et al., 2025), NDI (Cao and Yang, 2023), CIVE (Sunoj et al., 2025), RGRI (Chen et al., 2024a), VARI (Ramírez-Juidias and Antón, 2025), MGRVI (Feng et al., 2022), RGBRI (Liu et al., 2024a), GBRI (Sugara et al., 2025), IKAW (Liang et al., 2022), NGBDI (Le Saint et al., 2024), NDVI (Zheng et al., 2021b), RDVI (Liu et al., 2024a), NDRE (Liu et al., 2024a), NDCI (Mishra and Mishra, 2012), GNDVI (Liu et al., 2024a), MDD (Zha et al., 2020), EVI2 (MacDonald et al., 2025), NGI (Rossi et al., 2025), OSAVI (Liu et al., 2024a), TVI (Liu et al., 2024a), ENDI (Cao and Yang, 2023), TDVI (Putra et al., 2021), TCARI (Delegido et al., 2013), NDREI (Fu et al., 2024), LCI (Feng et al., 2022).

Detailed information on the vegetation indices, including their full names and formulas, is provided in **Supplementary Table A3**.

Texture features were extracted using the Gray-Level Co-occurrence Matrix (GLCM) method (Haralick et al., 1973). Based on the ROIs delineated in ENVI, each complete ROI was used as the fundamental GLCM calculation unit. GLCM statistics were calculated from the pixels within each ROI and summarized as one texture-descriptor vector for the corresponding ROI sample; texture values were not assigned to individual pixels using a moving-window procedure. Training and validation ROIs were processed separately, and texture descriptors from the same ROI were not shared between the two subsets. This procedural separation prevents direct reuse of ROI-level texture statistics, but it does not imply full geographical independence between nearby, distinct ROIs; the corresponding spatial limitation is described in Sections 2.2(4) and 4.3.

Preliminary experiments conducted exclusively on the training partition indicated that classification performance was similar when using 32-level and 64-level gray-scale quantization, whereas performance declined when 256 gray levels were adopted. Therefore, considering both feature representation capability and computational efficiency, a 32-level gray-scale quantization scheme was selected. The GLCM distance parameter was set to 5 pixels. To reduce sensitivity to directional variation, GLCMs were generated in four directions (0°, 45°, 90°, and 135°), and the resulting texture statistics were averaged across directions to obtain directionally invariant texture descriptors.

To avoid potential ROI-level information leakage, texture features were independently extracted from the training and validation ROI samples. The two datasets were processed separately, and no ROI information or texture statistics were shared between them. Finally, five texture features were selected for subsequent analyses, including Haralick correlation (harcor), entropy (entr), sum average (savg), cluster shade (cshade), and contrast (cont).

#### 2.3.3 Feature selection

To evaluate the effects of different feature selection paradigms on classification performance, two feature optimization strategies were designed in this study: fusion-before-selection and selection-before-fusion.

1. Fusion-before-selection: All features extracted from the RGB dataset (including spectral bands, vegetation indices, and texture features) and the multispectral dataset (including spectral bands, vegetation indices, and texture features) were directly combined to form a high-dimensional joint feature set. Feature selection algorithms were then applied to the fused feature space to identify the optimal feature subset.
2. Selection-before-fusion: Feature selection was performed independently on the RGB and multispectral datasets. First, the RGB dataset (including spectral bands, vegetation indices, and texture features) was separately optimized to obtain an RGB feature subset. Simultaneously, feature selection was conducted on the multispectral dataset to obtain a multispectral feature subset. Finally, the two selected feature subsets were merged and subjected to a second round of feature selection, yielding the final feature subset.

Under the two feature optimization strategies described above, the following three feature selection algorithms were consistently employed:

Successive Projections Algorithm (SPA) (Soares et al., 2013): SPA progressively selects variables based on vector projection principles by minimizing linear redundancy between newly selected features and previously selected ones. In this study, a supervised initialization strategy was adopted, where the feature with the highest mutual information was selected as the starting point of the search process. Feature selection was subsequently guided by the Fisher discriminative ability of projection residuals, thereby considering both feature independence and class separability.

Maximum Relevance Minimum Redundancy (mRMR) (Hanchuan et al., 2005): mRMR is a mutual information-based feature selection method that seeks to maximize the relevance between features and class labels while minimizing redundancy among features. An incremental search strategy was employed to progressively add features, aiming to achieve strong classification performance with a compact feature subset.

ReliefF (Spolaôr et al., 2013): ReliefF is an instance-based feature weighting algorithm that evaluates the discriminative contribution of each feature by comparing randomly sampled instances with their nearest neighbors from the same and different classes. This method is particularly effective in assessing the local discriminative capability of features.

The three feature selection algorithms were applied under both optimization strategies, resulting in six feature selection schemes that were subsequently used for input feature optimization in the classification models.

#### 2.3.4 Classification methods

Three machine learning models were employed for land-cover classification in this study: Support Vector Machine (SVM) (Mandal et al., 2022), K-Nearest Neighbors (KNN) (Zhuang et al., 2025), and Logistic Regression (LR) (Aspinall, 2002). SVM maps features into a high-dimensional space through a kernel function and constructs a maximum-margin hyperplane for classification. KNN classifies samples based on distance measurements and majority voting among the nearest neighbors. LR performs classification by modeling the relationship between feature variables and class probabilities.

Grid search combined with stratified five-fold cross-validation was used to optimize the key hyperparameters of each model. To ensure fair comparisons among different feature selection strategies and classification models, feature selection, hyperparameter optimization, and model training were conducted exclusively on the training dataset. Before model training, the selected features in the training dataset were standardized, and the same transformation was applied to the corresponding validation data. The primary analysis used a fixed random seed of 28 for the train–validation split and stratified five-fold cross-validation. To assess the sensitivity of the results to the particular data partition, an additional split-sensitivity analysis was performed using four alternative random seeds (12, 42, 225, and 3047). For each seed, the train–validation partition was regenerated, and the complete modeling procedure, including feature selection, five-fold cross-validation for hyperparameter optimization, model fitting, and independent validation, was repeated using the same experimental settings. Final classification performance was assessed using the corresponding independent validation dataset.

For SVM, C was searched over {40, 60, 80, 100, 120, 140} and the RBF kernel parameter γ over {scale, 0.5, 0.1, 0.01, 0.001}; the one-vs-one strategy was used for multiclass classification. For KNN, k was searched over {7, 9, 11, 13, 15}, using the Euclidean distance (Minkowski metric with p = 2) and uniform neighbor weights. For LR, C was searched over {0.01, 0.1, 1, 7, 9, 10, 100}, with L2 regularization, the SAGA solver, and a maximum of 50,000 iterations. The values 7 and 9 were retained because they frequently yielded high cross-validation accuracies during the initial exploratory search. No class weighting was applied to any classifier. The combination yielding the highest mean accuracy under five-fold cross-validation was selected. All analyses were implemented in Python 3.9.23 using scikit-learn 1.6.1, NumPy 1.26.4, SciPy 1.13.1, and pandas 2.3.3.

#### 2.3.5 Evaluation methods and experimental settings

##### 2.3.5.1 SHAP-based feature contribution analysis using RF

SHAP was used to quantify the global contributions of features to RF predictions (Lundberg and Lee, 2017). An RF model served as the explainer because it can represent nonlinear relationships and interactions among predictors. RF-SHAP was used only for exploratory feature analysis and not to interpret the final classification models. For feature *i*, the Shapley value is calculated as follows:


$$\phi_{i}=\sum_{S\subseteq F\setminus \{i\}}\;\frac{|S|!(|F|-|S|-1)!}{|F|!}[v(S\cup \{i\})-v(S)]$$

where $\phi_{i}$ denotes the Shapley value of feature *i*; *F* represents the complete feature set, and |F| denotes the total number of features. *S* is any subset of features that does not contain feature *i*, and |S| denotes the number of features in subset *S*. The function v(·) represents the expected prediction of the model for a specific feature combination. The term v(S∪{i})−v(S) represents the marginal contribution generated by adding feature *i* to subset *S*. By calculating the weighted sum of marginal contributions across all possible feature subsets, the contribution of each feature to the classification task can be quantitatively evaluated.

##### 2.3.5.2 Classification accuracy assessment

Classification performance was evaluated using Overall Accuracy (OA), Kappa Coefficient, Producer’s Accuracy (PA), User’s Accuracy (UA), and F1-score. The corresponding calculation formulas are presented in **Table 3**.

**Table 3:** Evaluation metrics.

Indicator	Calculation formula
Overall Accuracy (OA)	$OA=\frac{\sum_{i=1}^{k}n_{ii}}{N}$
Kappa Coefficient	$Kappa=\frac{P_{o}-P_{e}}{1-P_{e}}$
Producer’s Accuracy (PA)	$PA_{i}=\frac{n_{ii}}{n_{i+}}$
User’s Accuracy (UA)	$UA_{i}=\frac{n_{ii}}{n_{+i}}$
F1-score	$F1_{i}=\frac{2\cdot PA_{i}\cdot UA_{i}}{PA_{i}+UA_{i}}$

*N*, total number of samples; *k*, number of categories; $n_{ii}$, number of correctly classified samples for class *i*; $P_{o}$, equivalent to OA; $P_{e}=\frac{\sum_{i=1}^{k}(n_{i+}\cdot n_{+i})}{N^{2}}$; $n_{i+}$, total number of reference samples in class i; $n_{+i}$, total number of samples predicted as class i.

##### 2.3.5.3 Experimental settings

For the SPA, mRMR, and ReliefF feature selection methods, candidate feature ranking lists were first generated according to the evaluation criteria of each algorithm. The stopping thresholds were determined through preliminary experiments conducted exclusively on the training partition, without accessing the validation data. These experiments focused on controlling the number of retained candidate features rather than maximizing validation performance. Based on the training-only experiments, the thresholds were empirically set to retain a moderate-sized candidate feature pool, thereby avoiding excessive elimination of potentially informative features while excluding a large proportion of low-contribution features. Specifically, for SPA, the Fisher discriminative score was adopted as the stopping criterion, and the search process was terminated when the discriminative contribution of a candidate feature fell below 0.007. For mRMR, the search process was terminated when the mRMR score (relevance minus redundancy) of the best candidate feature fell below 0.105. For ReliefF, features with weights below 0.09 were discarded.

The resulting candidate feature subsets were then subjected to progressive feature inclusion according to the feature ranking order to determine the final candidate subsets for classification. After the one-stage or two-stage feature selection procedure was completed, the selected feature subsets were fixed. The optimal number of features and model hyperparameters were subsequently determined using five-fold cross-validation exclusively within the training partition. The resulting models and selected feature numbers were then fixed and evaluated on the external validation set, which was not used during threshold calibration, feature selection, feature-number selection, or hyperparameter optimization. To further assess the sensitivity of the selected thresholds, a local sensitivity analysis was conducted using the training partition only. For each feature-selection method, the adopted threshold was perturbed over a small range around the selected value, while the number of features used for classification was fixed at the value adopted in the main analysis. Classification performance was evaluated using five-fold cross-validation within the training partition. The results are provided in **Supplementary Table A4**.

## 3 Results

### 3.1 Single-source feature analysis

#### 3.1.1 Comparison of feature importance

##### 3.1.1.1 Feature importance assessment

RF-SHAP identified the ten most influential features in each data source (**Figure 3**; complete rankings in **Supplementary Table A2**). RGB rankings were dominated by visible-light vegetation indices, with B_RGB the only original band among the top ten. In the multispectral set, R_MS ranked first (10.16%), followed mainly by indices containing near-infrared or red-edge information, including NDCI, RDVI, and EVI2. No texture metric appeared in either top-ten list, indicating a larger global contribution from spectral and index features in the RF model. This does not imply that texture features were uninformative; their complementary value was assessed through correlation and feature-selection analyses.

**Figure 3:**
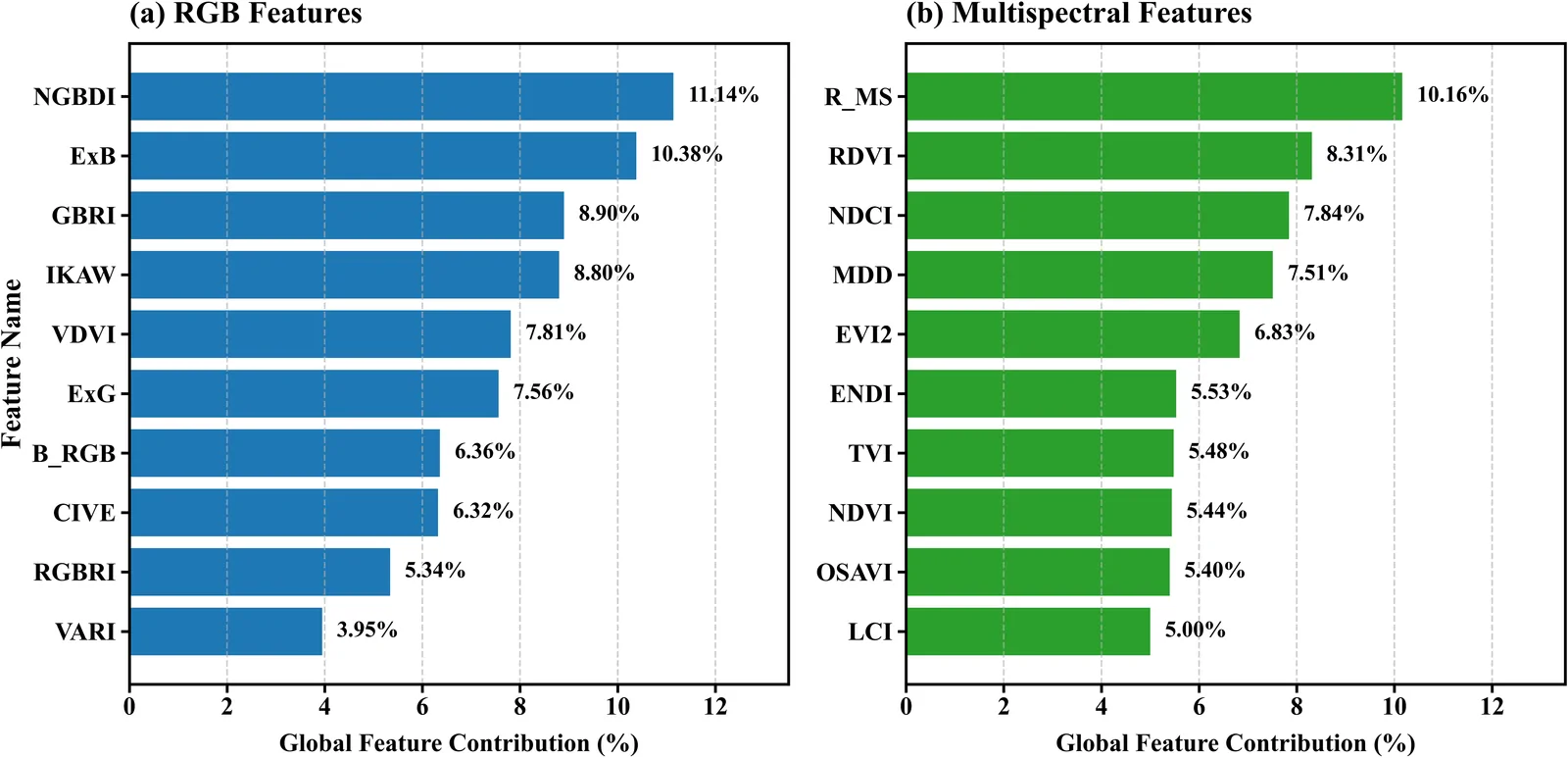
Top ten features ranked by RF-SHAP importance in the RGB and multispectral datasets: **(a)** RGB Features; **(b)** Multispectral Features. Statistical significance is indicated by * (p < 0.05) and ** (p < 0.01).

##### 3.1.1.2 Feature separability assessment

The top ten RF-SHAP features from each data source were evaluated using the Kruskal–Wallis test and Dunn’s *post hoc* comparisons (**Figure 4**; **Supplementary Table A1**). All features differed among classes (p < 0.001), but separability varied. RGB indices such as NGBDI, ExG, RGBRI, and VARI distinguished bare land from vegetation but showed overlap among Ac, As, and Cm. Multispectral features provided clearer separation: R_MS distinguished bare land, while RDVI, NDVI, and EVI2 showed more consistent gradients among vegetation classes.

**Figure 4:**
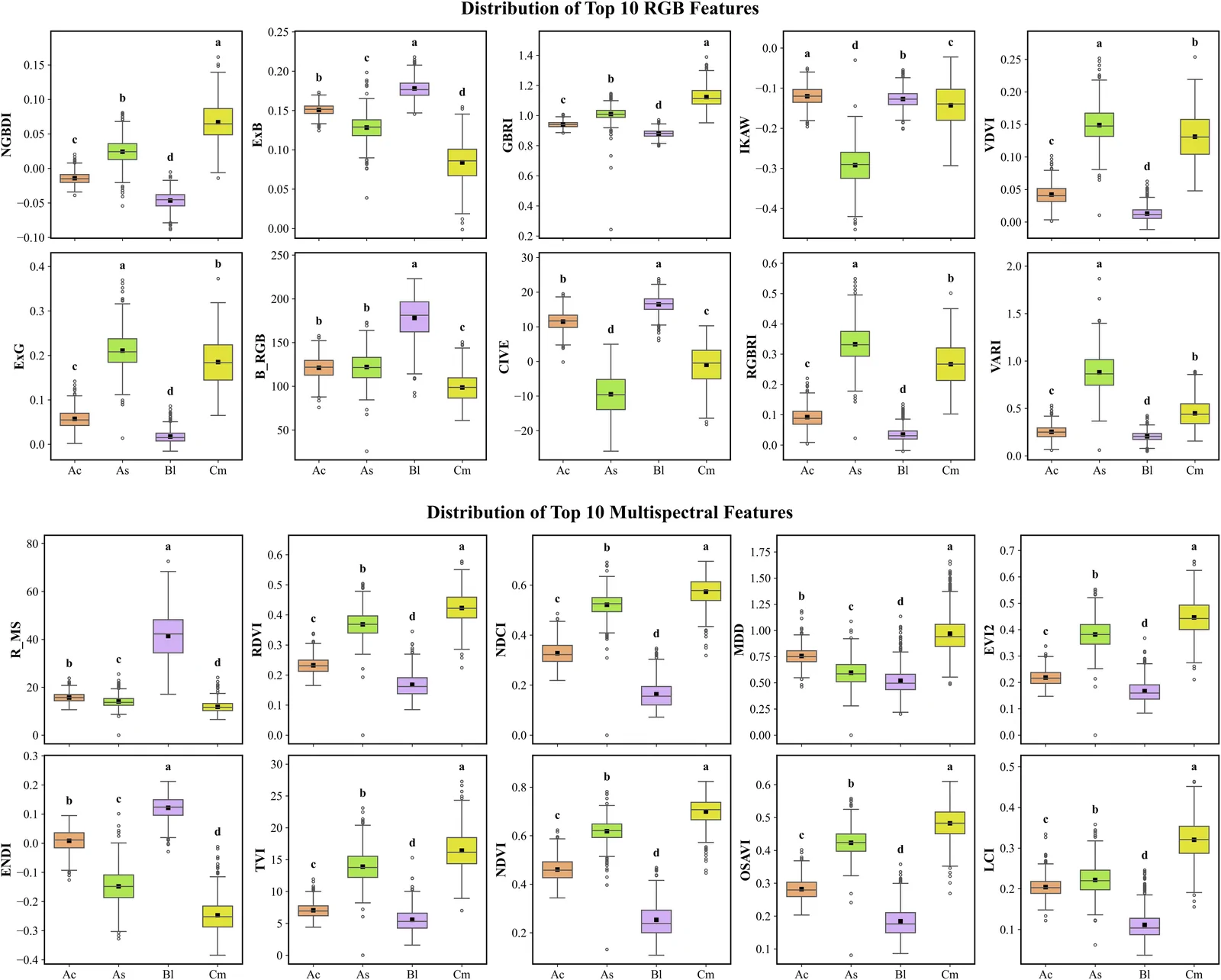
Distribution characteristics of the top 20 RF-SHAP-ranked features across the four land-cover classes. The lowercase letters indicate statistical grouping based on the Kruskal–Wallis test followed by Dunn’s post hoc pairwise comparisons with Holm adjustment. Within each feature, the group with the highest mean value is assigned the letter “a”; groups sharing the same letter are not significantly different, whereas groups with different letters differ significantly (adjusted p < 0.05).

#### 3.1.2 Inter-feature correlation analysis

Spearman’s rank correlations were calculated within the multispectral and RGB feature sets. Statistical significance is indicated by * (p < 0.05) and ** (p < 0.01).

(1) Correlation Analysis of the Multispectral Feature Set

The multispectral correlation matrix (**Figure 5**) showed strong correlations among vegetation indices such as NDVI, OSAVI, and GNDVI (mostly |r| > 0.8). R_MS was also strongly correlated with many indices. By contrast, texture features such as harcor_ms and cont_ms were weakly correlated with most other features (generally |r| < 0.4).

**Figure 5:**
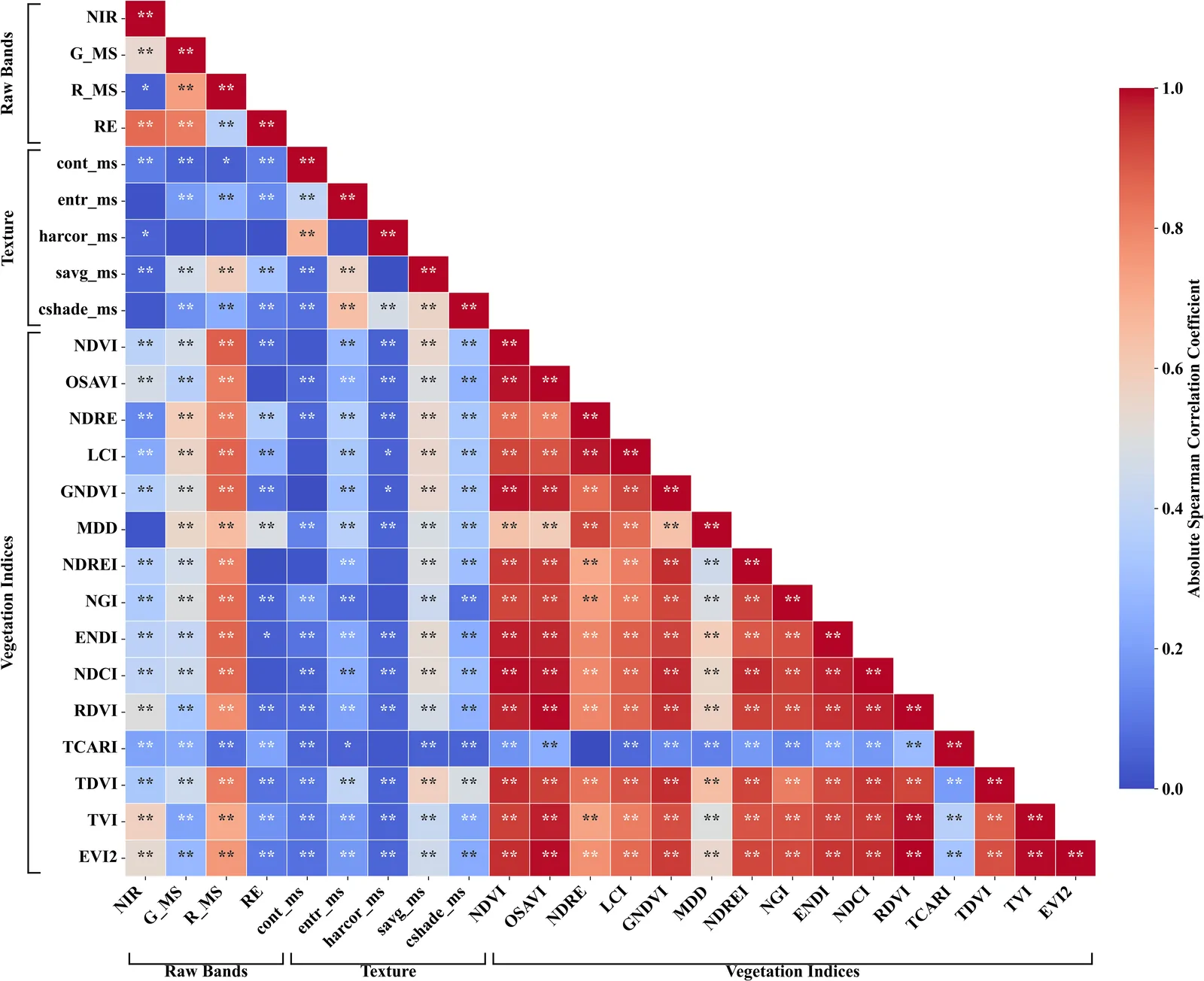
Spearman correlation matrix of the multispectral feature set.

(2) Correlation Analysis of the RGB Feature Set

The RGB correlation matrix showed a similar pattern (**Figure 6**). R_RGB was strongly correlated with most visible-light indices, whereas NDI, IKAW, and GBRI were less correlated with the dominant index group. Texture features, including cont_rgb, harcor_rgb, and entr_rgb, were weakly correlated with spectral bands and vegetation indices.

**Figure 6:**
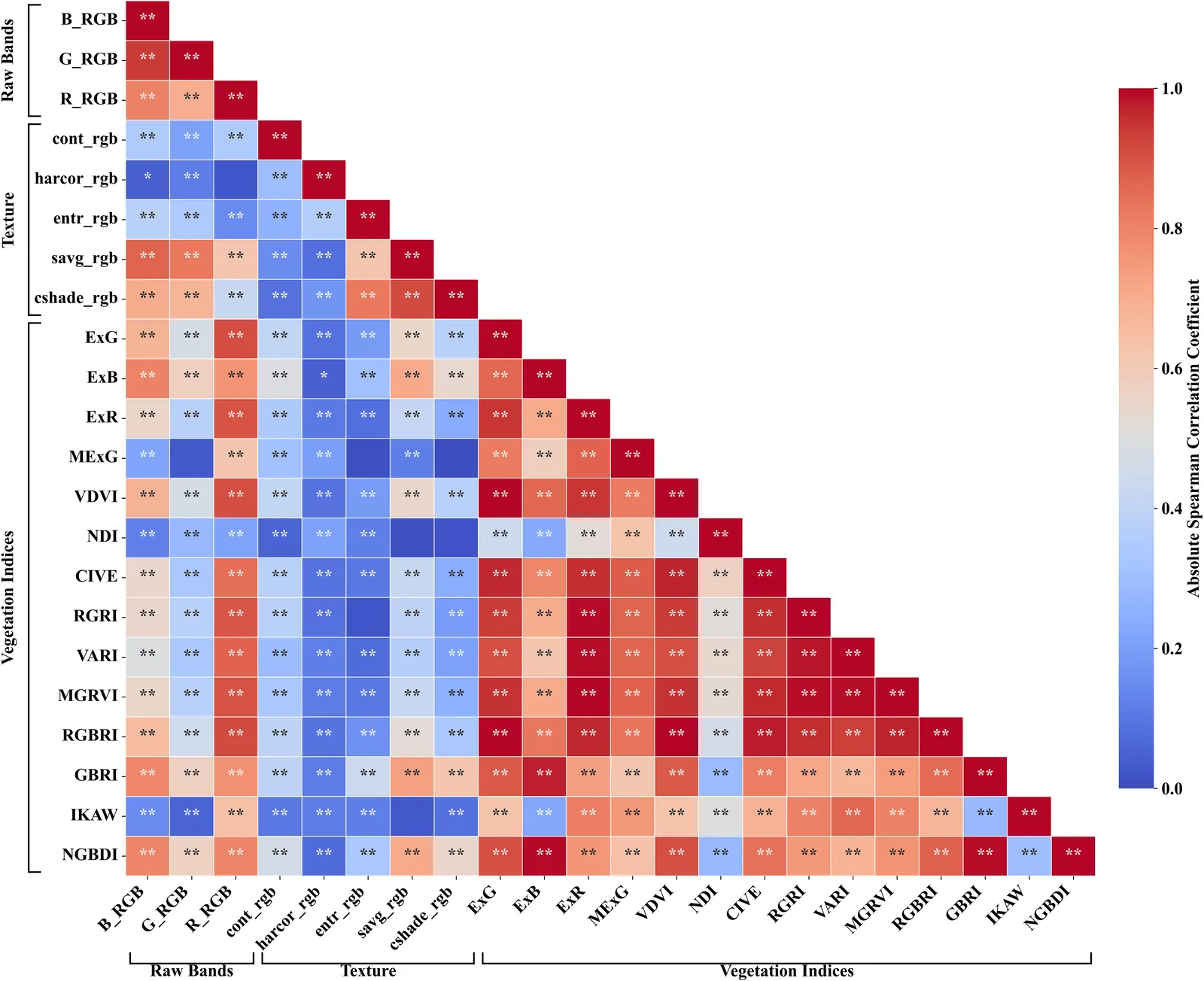
Spearman correlation matrix of the RGB feature set.

Both feature sets therefore contained substantial redundancy among vegetation indices, while texture features provided comparatively independent spatial information.

#### 3.1.3 Single-source feature selection and classification evaluation

**Figure 7** summarizes the features retained from the RGB and multispectral datasets. The algorithms showed moderate agreement: NGBDI was selected by all methods for RGB data (blue), and NDVI and LCI were selected by all methods for multispectral data (orange). VARI and NGI were also retained repeatedly, indicating stable discriminative value across selection criteria.

**Figure 7:**
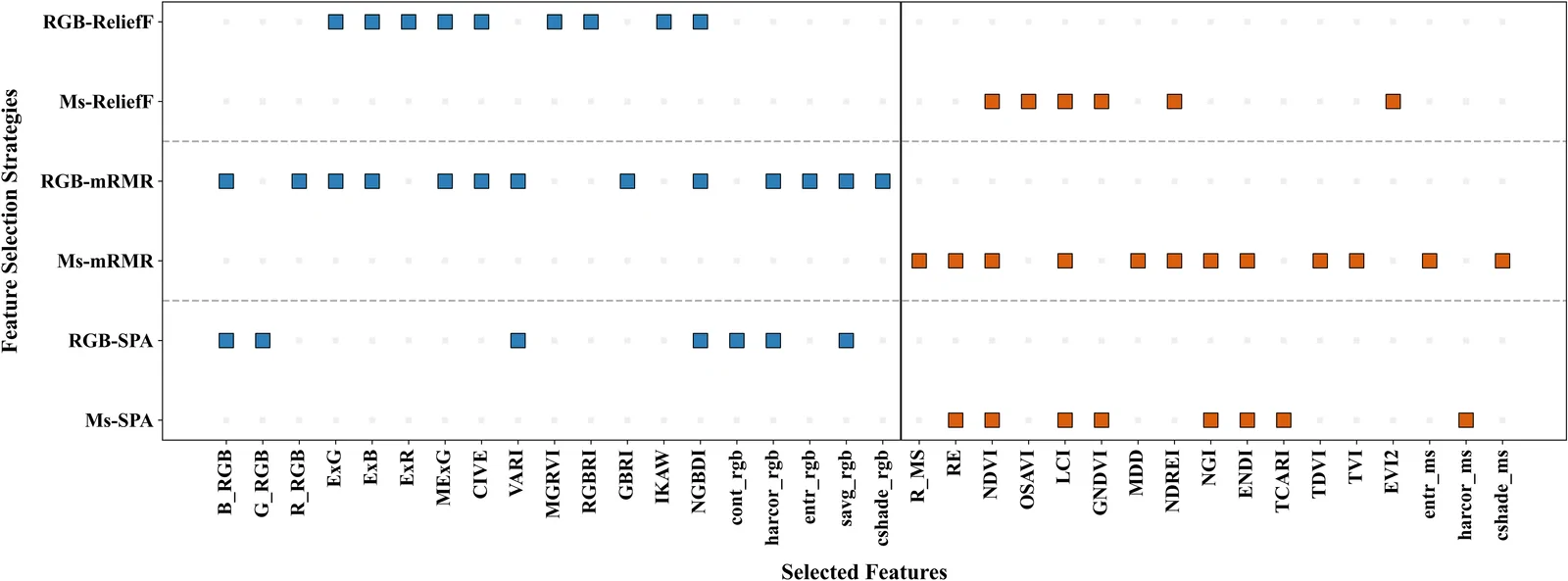
Feature selection results for the RGB and multispectral datasets.

All three methods preferentially retained vegetation indices (**Figure 8**). For RGB data, ReliefF selected only indices, whereas SPA and mRMR also retained texture features, indicating complementary value from canopy structure. Multispectral subsets consisted mainly of indices and original bands, with few texture features retained; thus, spectral information remained the principal basis for class discrimination.

**Figure 8:**
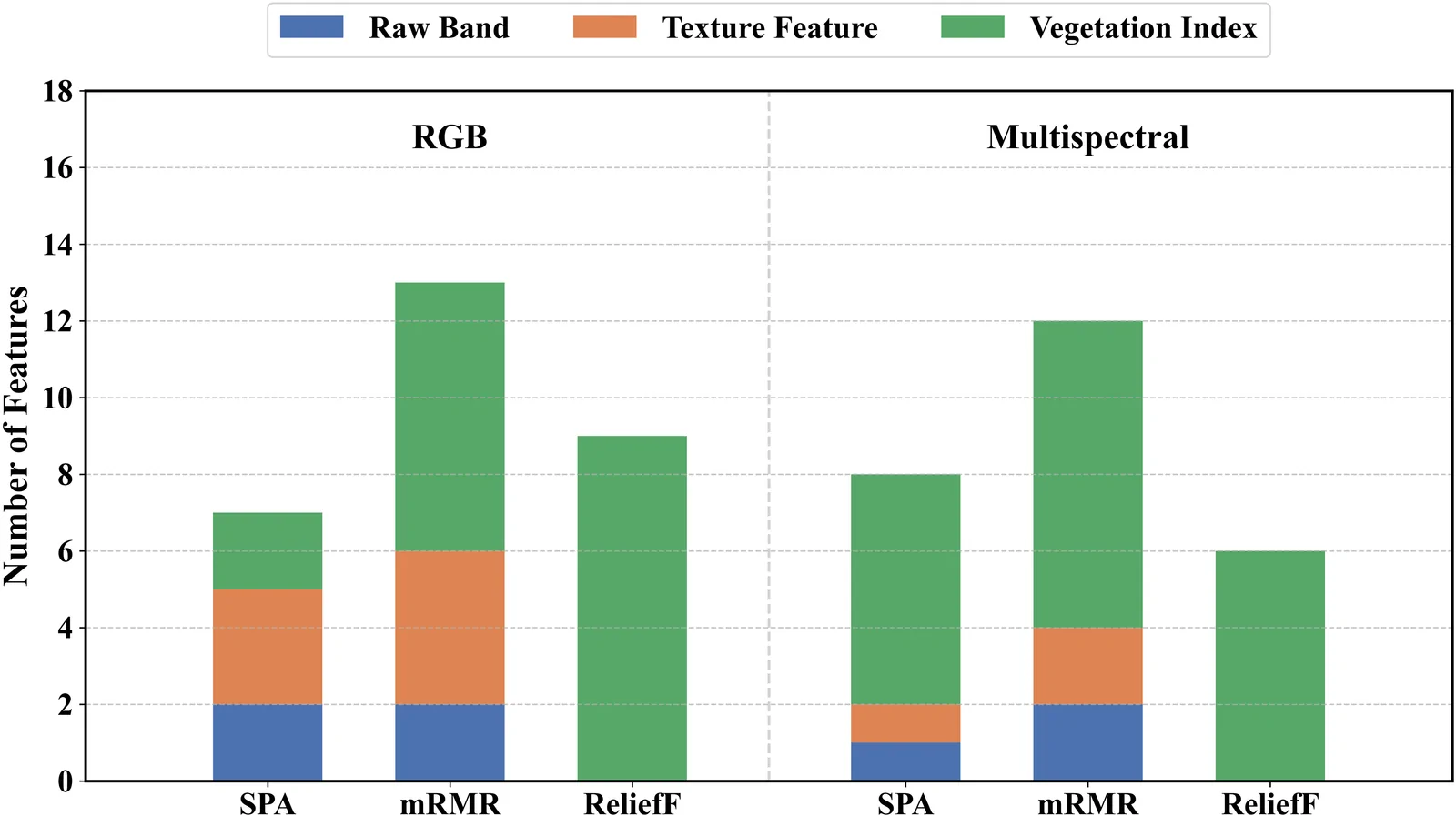
Composition of selected feature types for the RGB and multispectral datasets.

Most reduced feature sets matched or exceeded the full-feature baselines (**Figure 9**). For multispectral data, SPA+SVM retained eight features and achieved an OA of 87.37% and Kappa of 83.16%, compared with 86.74% and 82.32% for the 24-feature baseline. ReliefF retained six features with comparable performance (OA 86.87%; Kappa 82.48%). For RGB data, ReliefF+SVM retained nine features and improved OA from 88.13% to 89.02% and Kappa from 84.17% to 85.35%. These results show that compact, nonredundant subsets can maintain or improve classification performance.

**Figure 9:**
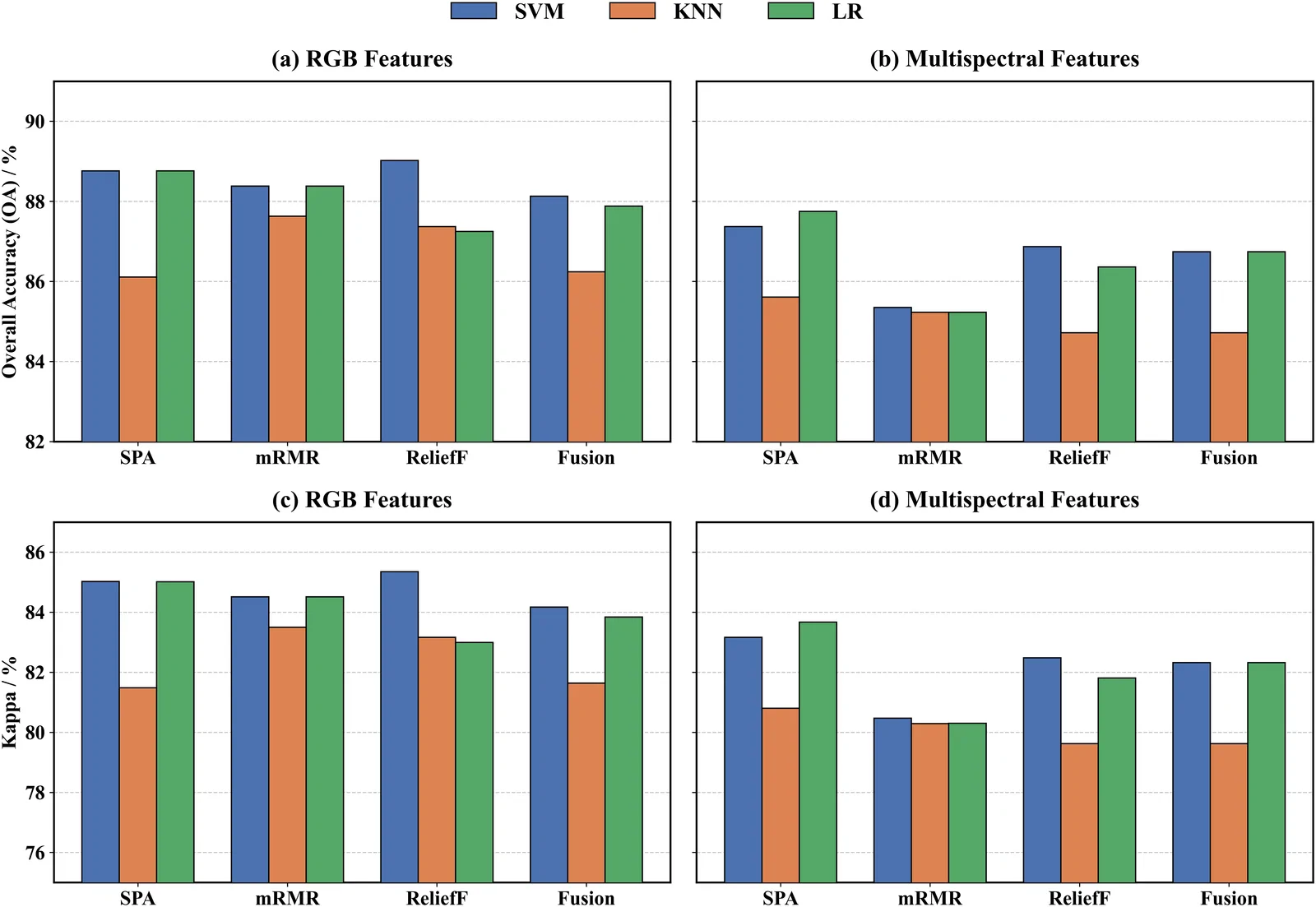
Comparison of OA and Kappa coefficients obtained using different feature selection methods and classifiers for the RGB and multispectral datasets: **(a)** overall accuracy (OA) for RGB features; **(b)** overall accuracy (OA) for multispectral features; **(c)** Kappa coefficient for RGB features; and **(d)** Kappa coefficient for multispectral features.

### 3.2 Evaluation of multi-source feature fusion

#### 3.2.1 Cross-source feature complementarity analysis

The cross-source Spearman matrix (**Figure 10**) revealed strong correlations (|r| > 0.8) between many multispectral and RGB vegetation indices, including NDVI, NDREI, and EVI2 versus ExB, VDVI, and CIVE. In contrast, NIR, RE, and multispectral texture features were weakly correlated (generally |r| < 0.4) with RGB color and texture features. Thus, redundancy was concentrated among indices, whereas cross-category feature pairs offered greater complementarity.

**Figure 10:**
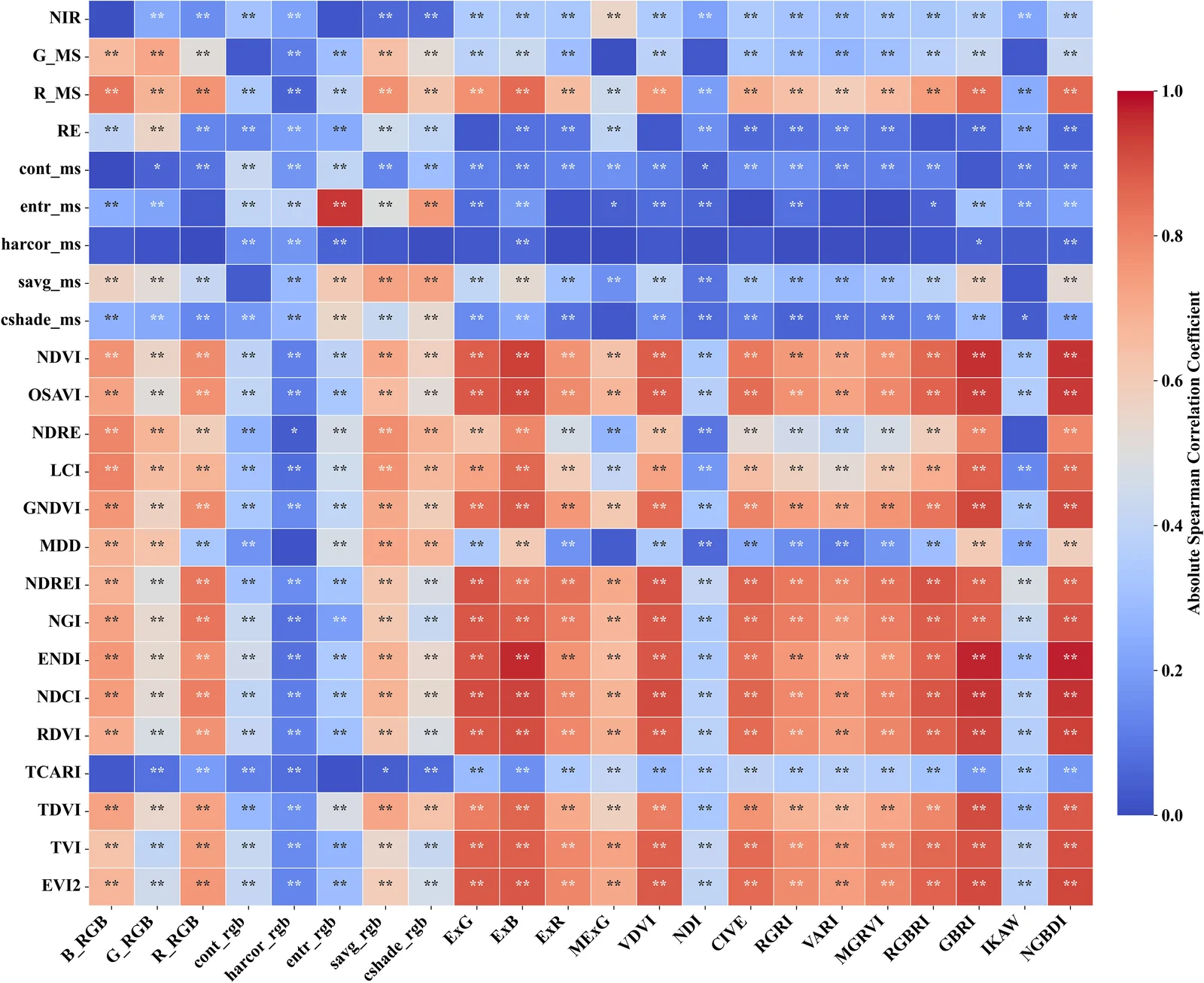
Spearman absolute correlation matrix of cross-source features.

#### 3.2.2 Performance evaluation of different fusion strategies

##### 3.2.2.1 Comparison of feature selection results

**Figure 11** compares feature subsets generated under the one-stage (Os) and two-stage (Ts) strategies (RGB: blue; Multispectral: orange). Os produced more compact subsets (6–10 features), dominated by vegetation indices and a few texture metrics. Ts retained 6–12 features and, for some algorithms, preserved more original bands. SPA was particularly sensitive to fusion order: both strategies retained NDVI, ENDI, NGI, savg_rgb, and harcor_rgb, whereas Ts-SPA additionally retained VARI, R_MS, B_RGB, GNDVI and TCARI. ReliefF produced more similar subsets across strategies. These patterns suggest that source-wise screening can preserve a broader range of complementary feature types.

**Figure 11:**
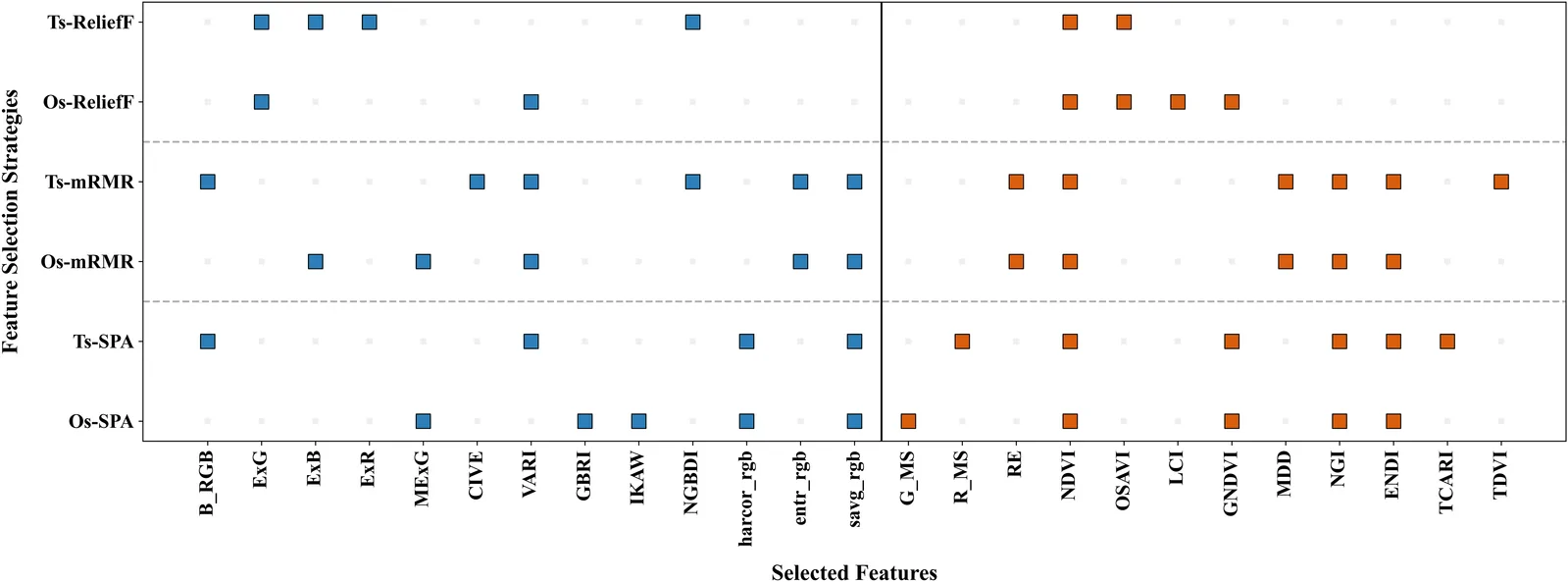
Comparison of feature subsets selected by different feature selection methods under the one-stage (Os) and two-stage (Ts) fusion strategies.

##### 3.2.2.2 Accuracy comparison

**Table 4** compares Direct Fusion, Os, and Ts across classifiers. Direct Fusion with LR achieved an OA of 90.91% and a Kappa of 87.88%, whereas Ts-SPA+LR yielded the highest observed OA (91.54%) and Kappa (88.72%) using only 10 features. However, the 0.63-percentage-point OA improvement of Ts-SPA+LR over Direct-LR was not statistically significant (**Table 5**; raw McNemar’s p = 0.4995; 95% bootstrap CI for ΔOA: −0.88 to 2.15 percentage points). Consistently, the corresponding Kappa difference (+0.84 percentage points) also had a 95% bootstrap CI spanning zero (−1.16 to 2.85 percentage points). These results indicate that the small numerical advantage of Ts-SPA+LR over Direct-LR may be attributable to sampling variability. After Holm correction for multiple comparisons, Ts-SPA+LR showed significantly higher accuracy than Ts-mRMR+LR (ΔOA = 3.03 percentage points, adjusted p = 0.0154) and Ts-ReliefF+LR (ΔOA = 3.91 percentage points, adjusted p = 0.0012). For SVM, Ts-SPA achieved a higher OA than Direct Fusion (+1.26 percentage points), but the difference was not statistically significant (adjusted p = 0.6606). In contrast, Ts-SPA significantly reduced KNN performance relative to Direct Fusion (−2.78 percentage points, adjusted p = 0.0200). These results indicate that the effect of fusion strategy depended on the feature selection algorithm and classifier, and that Ts-SPA+LR achieved the highest observed OA and Kappa among the evaluated combinations but was not statistically superior to the Direct-LR baseline. To assess the sensitivity of this result to the particular train–validation partition, the LR-based analysis was additionally repeated with four alternative random seeds. As shown in **Supplementary Table A5**, Ts-SPA+LR remained highly competitive across the five partitions, with OA values ranging from 91.29% to 91.54% and outperforming Direct-LR in four of the five splits. In the remaining split, the difference was small, with Direct-LR achieving 91.92% versus 91.29% for Ts-SPA+LR. Thus, the exact numerical advantage varied across partitions, but the strong performance of Ts-SPA+LR was not solely attributable to the original seed-28 split.

**Table 4:** Classification performance of different fusion strategies and feature selection methods.

Strategy	Method	Dim	SVM		KNN		LR	
			OA (%)	Kappa (%)	OA (%)	Kappa (%)	OA (%)	Kappa (%)
Direct	Fusion	46	90.03	86.70	90.66	87.54	90.91	87.88
Os	SPA	10	90.03	86.70	89.14	85.52	90.15	86.87
	mRMR	10	89.02	85.35	89.77	86.36	89.02	85.35
	ReliefF	6	90.91	87.87	88.64	84.84	90.15	86.86
Ts	SPA	10	**91.29**	**88.38**	87.88	83.84	**91.54**	**88.72**
	mRMR	12	89.77	86.36	89.39	85.85	88.51	84.68
	ReliefF	6	89.65	86.19	**90.03**	**86.69**	87.63	83.50

Os and Ts denote the one-stage and two-stage fusion strategies, respectively; Dim represents the dimensionality of the selected feature subset. Bold values ​​indicate the highest OA and Kappa achieved by each classifier among the feature-selection methods.

**Table 5:** Pairwise comparison of classification performance using McNemar’s test and paired bootstrap confidence intervals.

Comparison	ΔOA(%)	McNemar p-value	Holm-adjusted p-value	95% CI for ΔOA (%)	ΔKappa(%)	95% CI for ΔKappa (%)
Ts-SPA-LR vs Direct-LR	+0.63	0.4995	0.6606	-0.88, 2.15	+0.84	-1.16, 2.85
Ts-SPA-SVM vs Direct-SVM	+1.26	0.1101	0.6606	-0.13, 2.65	+1.69	-0.16, 3.55
Ts-SPA-KNN vs Direct-KNN	-2.78	0.0020	**0.0200**	-4.55, -1.14	-3.70	-6.05, -1.52
Ts-SPA-SVM vs Os-SPA-SVM	+ 1.26	0.0871	0.6097	0.00, 2.65	+1.68	0.00, 3.53
Ts-SPA-KNN vs Os-SPA-KNN	-1.26	0.1101	0.6606	-2.65, 0.13	-1.68	-3.54, 0.17
Ts-SPA-LR vs Os-SPA-LR	+1.39	0.0432	0.3888	0.25, 2.65	+1.85	0.33, 3.54
Ts-SPA-SVM vs Ts-mRMR-SVM	+1.51	0.1336	0.6606	-0.25, 3.28	+2.02	-0.34, 4.41
Ts-SPA-KNN vs Ts-mRMR-KNN	-1.52	0.1550	0.6606	-3.41, 0.38	-2.02	-4.57, 0.50
Ts-SPA-LR vs Ts-mRMR-LR	+3.03	0.0014	**0.0154**	1.26, 4.92	+4.05	1.70, 6.52
Ts-SPA-SVM vs Ts-ReliefF-SVM	+1.64	0.1174	0.6606	-0.25, 3.54	+2.19	-0.32, 4.73
Ts-SPA-KNN vs Ts-ReliefF-KNN	-2.15	0.0532	0.4256	-4.17, -0.13	-2.86	-5.56, -0.18
Ts-SPA-LR vs Ts-ReliefF-LR	+3.91	0.0001	**0.0012**	1.89, 5.93	+5.22	2.55, 7.93

ΔOA and ΔKappa denote the differences in overall accuracy and Kappa, respectively, expressed in percentage points. McNemar’s test was based on paired predictions, and Holm-adjusted p-values were calculated for all 12 pairwise comparisons. 95% confidence intervals were estimated from 10,000 paired bootstrap resamples. Bold values indicate Holm-adjusted p < 0.05. Values in **Table 5** were calculated directly from the original prediction results; therefore, minor discrepancies may occur compared with the values reported in **Table 4**.

##### 3.2.2.3 Visual comparison

**Figure 12** provides a qualitative visual comparison of the classification maps produced by the different classification schemes. In several representative areas, the SVM- and LR-based maps appeared to contain fewer visually fragmented regions than the KNN-based maps, while the Ts-SPA maps appeared spatially more coherent than those produced by several other feature-selection methods. The Ts-SPA maps also showed visually sharper class transitions and fewer apparent isolated pixels in some representative areas. These observations are based solely on visual inspection and are intended to illustrate qualitative differences in map appearance. Because no object-level or patch-level spatial metrics were evaluated, they should not be interpreted as quantitative evidence of reduced fragmentation, improved boundary accuracy, or enhanced spatial continuity.

**Figure 12:**
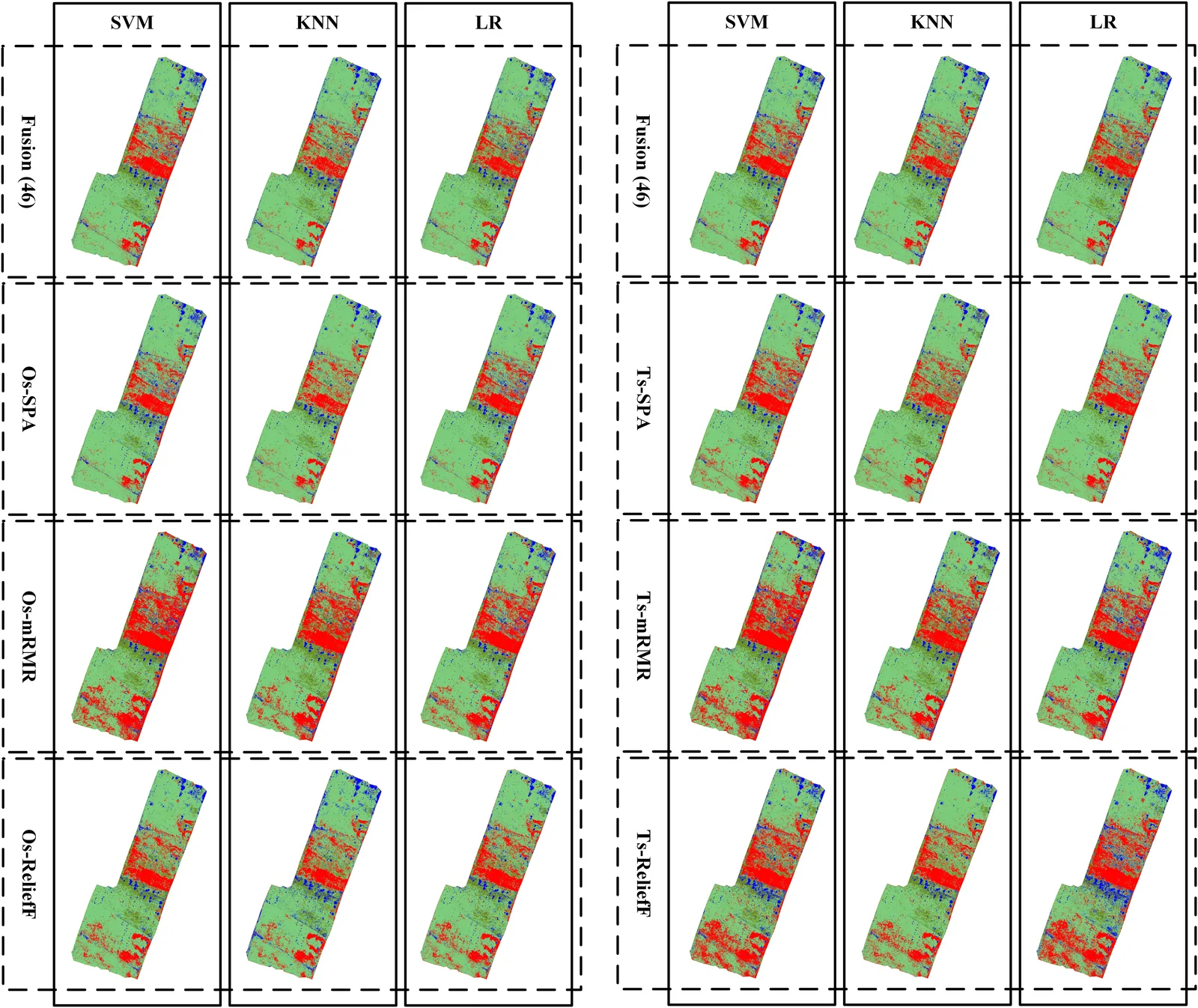
Comparison of classification maps produced by different fusion strategies and classifiers. Bare land (red), Caragana microphylla (blue), Artemisia scoparia (gray-green), and Agropyron cristatum (green).

### 3.3 Evaluation of the optimal classification model

#### 3.3.1 Classification accuracy and error analysis

Ts-SPA selected 10 features (NDVI, VARI, R_MS, ENDI, harcor_rgb, savg_rgb, B_RGB, NGI, GNDVI, and TCARI), which were used in the LR model. The model achieved 91.54% OA and a Kappa coefficient of 88.72% (**Table 6**). Artemisia scoparia, bare land, and Caragana microphylla had PA values of 94.00%, 94.92%, and 93.85%, respectively, with F1-scores of 93.53%, 90.55%, and 94.57%. Caragana microphylla had the highest UA (95.31%). Agropyron cristatum was the least accurate class (PA 83.50%; F1-score 87.44%): 26 samples were classified as bare land, and eight Caragana microphylla samples were classified as Agropyron cristatum. These errors likely reflect low vegetation cover and spectral similarity near shrub–grass boundaries.

**Table 6:** Confusion matrix corresponding to the highest overall accuracy.

Class	Ac	As	Bl	Cm	Total	PA (%)
Ac	167	7	26	0	200	83.50
As	7	188	3	2	200	94.00
Bl	0	3	187	7	197	94.92
Cm	8	4	0	183	195	93.85
UA(%)	91.76	93.07	86.57	95.31		
F1(%)	87.44	93.53	90.55	94.57		
	OA (%): 91.54 Kappa (%): 88.72					

#### 3.3.2 Visual comparison of classification results in typical sites

**Figure 13** compares Os-SPA+LR and Ts-SPA+LR at three representative sites. At Site 1, both models identified the main Caragana microphylla patches, while the Ts-SPA+LR map showed fewer visually apparent boundary discrepancies, although some internal fragmentation remained. At Site 2, the Ts-SPA+LR result appeared more consistent with the RGB image in areas of exposed soil, while both models showed local confusion between bare land and Agropyron cristatum. At Site 3, the Ts-SPA+LR result appeared to show less local confusion between Caragana microphylla and Artemisia scoparia, with somewhat clearer shrub boundaries. Overall, the Ts-SPA+LR map showed some visually apparent differences in complex transition zones relative to Os-SPA+LR, broadly consistent with the differences observed in the quantitative classification results. Because no object-level or patch-level spatial accuracy metrics were calculated, these observations are qualitative and should be interpreted as differences in map appearance rather than quantitative evidence of improved boundary accuracy, reduced fragmentation, or superior spatial coherence.

**Figure 13:**
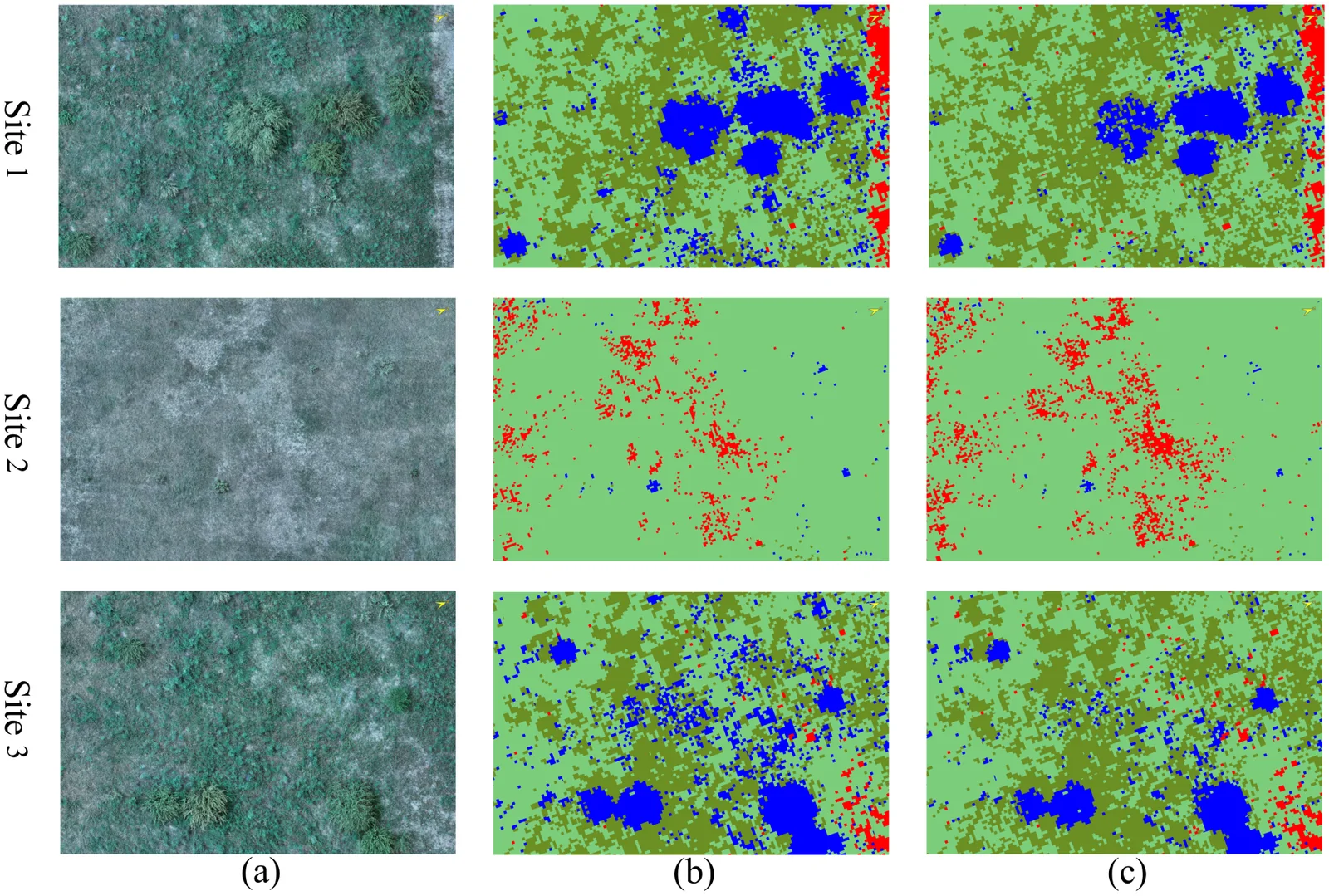
Comparison of classification results in three representative sites: **(a)** true-color RGB image; **(b)** Os+SPA+LR (10 features); and **(c)** Ts+SPA+LR (10 features). Red represents bare land, blue represents Caragana microphylla, green represents Agropyron cristatum, and gray-green represents Artemisia scoparia.

## 4 Discussion

### 4.1 Feature analysis

According to the RF-SHAP analysis, the red band (R_MS), NDCI, and RDVI from the multispectral set, together with NGBDI and ExB from the RGB set, showed the highest mean absolute SHAP values, indicating relatively large contributions to the predictions of the RF model (**Figure 3**). The Kruskal–Wallis analysis further confirmed significant interclass differences for these features (**Figure 4**). RGB-based vegetation indices such as NGBDI and ExG showed clear differences between bare land and vegetated classes, whereas multispectral indices including NDCI, RDVI, NDVI, and EVI2 showed greater differentiation among the three vegetation types, potentially because the near-infrared and red-edge bands are sensitive to differences in canopy biochemical and structural characteristics (Jitendra and Pant, 2024; Liu et al., 2024b).

The RF-SHAP ranking and the feature-selection results showed partial overlap, but they should not be interpreted as equivalent measures of feature relevance. NGBDI (RGB), NDVI, and LCI (multispectral), for example, were retained by all three selection algorithms (**Figure 7**), reflecting consistent selection across methods. In contrast, several texture features with relatively low RF-SHAP contributions, notably harcor_rgb and savg_rgb, were retained by SPA and mRMR. The correlation analysis (**Figures 5**, **6**) provides a possible explanation for this pattern: vegetation indices within each source were often strongly intercorrelated (Spearman |r| approaching 1.0), whereas texture features exhibited consistently weak correlations with spectral and vegetation-index features. Thus, redundancy-reducing selection algorithms could retain representative features from highly correlated index groups while also preserving texture variables that provide complementary information. This suggests that texture features may capture aspects of spatial heterogeneity and neighborhood structure that are less directly represented by spectral responses. Such complementarity between spectral and textural information has also been reported in high-resolution classification studies (Bazzo et al., 2024; Zhao et al., 2026) and supports the potential complementarity of texture features even when their univariate importance scores appear low.

The ecological and spectral characteristics of the final Ts-SPA feature subset provide a plausible interpretation of its classification performance. The selected multispectral features, including R_MS, NDVI, GNDVI, ENDI, NGI, and TCARI, provide complementary information on vegetation spectral responses, greenness, and canopy pigment characteristics. NDVI and GNDVI characterize vegetation-related differences in spectral reflectance, while NGI provides a normalized representation of green reflectance relative to the NIR, green, and red-edge signals, and ENDI provides additional information on red–green spectral contrast (Gitelson et al., 1996; Liu et al., 2019). TCARI further provides information related to canopy pigment characteristics (Haboudane et al., 2002). The RGB-derived features, including VARI, B_RGB, harcor_rgb, and savg_rgb provide complementary information on visible-band color and vegetation greenness, which may help distinguish vegetated surfaces from bare land and capture subtle differences among vegetation types (Gitelson et al., 2002; Anzar et al., 2025). Among these, harcor_rgb and savg_rgb may provide complementary information on canopy heterogeneity and spatial arrangement (Bazzo et al., 2024; Zhao et al., 2026). Collectively, these features represent vegetation spectral properties, visible-band characteristics, and canopy spatial structure, providing complementary cues for separating bare land, shrub, and herbaceous vegetation.

### 4.2 Analysis of feature selection strategies

The cross-source correlation matrix (**Figure 10**) revealed structured multicollinearity among vegetation indices across the RGB and multispectral datasets (many pairs with |r| > 0.8), highlighting a central challenge for multi-source fusion. The one-stage (Os) and two-stage (Ts) strategies addressed this redundancy through distinct mechanisms. Os tended to preferentially retain vegetation indices within the fused feature space, limiting the opportunity for original spectral bands to be selected. In contrast, Ts performed within-source screening before cross-source refinement, allowing additional spectral bands (e.g., B_RGB, R_MS) to be retained alongside core vegetation indices and texture features. This suggests that decoupling the two steps may better preserve complementary information from different feature types.

The response to fusion strategy varied considerably across selection algorithms. SPA was particularly sensitive: Ts-SPA retained the key variables shared with Os-SPA (NDVI, ENDI, NGI, savg_rgb, harcor_rgb) while additionally incorporating VARI, R_MS, B_RGB, GNDVI and TCARI, yielding the highest observed performance with the LR classifier (OA = 91.54%, Kappa = 88.72%). Relative to Direct Fusion, Ts-SPA+LR reduced feature dimensionality by 78.26% (from 46 to 10 features) while maintaining a comparable classification accuracy, with an observed OA difference of +0.63 percentage points that was not statistically significant. This substantial reduction suggests that a considerable proportion of the original fused features provided redundant or limited additional information for classification. Notably, the retained features represented complementary information from vegetation indices, spectral bands, and texture descriptors, indicating that feature selection preserved major discriminative information while substantially reducing the number of predictors. A more compact feature subset can also simplify the classification workflow and improve feature interpretability by reducing the number of variables that need to be extracted, processed, and considered during model development. This benefit is particularly relevant to UAV-based remote sensing workflows, in which multisource imagery can generate numerous potentially redundant features. Previous studies have likewise reported that feature selection can substantially reduce the feature set while improving computational efficiency without compromising classification performance (Zhou et al., 2018). In addition, the results indicate that no single fusion strategy is universally superior: effectiveness depends on the interaction between the fusion paradigm and the selection algorithm. This finding is consistent with recent work showing that feature optimization improves multisource classification in similar grassland settings (Chen et al., 2024b; Guan et al., 2025), and it further suggests that workflow design should consider the coupling between fusion order and algorithm choice rather than treating them as independent decisions.

### 4.3 Application value and research limitations

The proposed workflow showed potential for fine-scale classification of the target grassland land-cover classes, but several limitations remain.

In sparsely vegetated areas with low vegetation cover, soil background signals may reduce the spectral separability of Agropyron cristatum (PA = 83.50%, UA = 91.76%), thereby increasing confusion between this class and bare land. Besides, the multispectral imagery was reconstructed in DJI Terra with illumination compensation based on onboard sunlight-sensor records, image metadata, and manufacturer-provided calibration parameters, whereas the RGB-derived features were calculated from digital color values (0–255). The multispectral variables therefore represent irradiance-compensated relative spectral responses, while the RGB variables represent relative color and intensity information. This difference in physical meaning may affect the relative contributions of the two feature sources and limits the cross-scene transferability of RGB-derived variables under changing illumination conditions. In shrub–grass transitional zones with relatively high spectral similarity (e.g., Site 3 in **Figure 13**), local misclassified patches between Caragana microphylla and Artemisia scoparia were still observed, resulting in a typical “salt-and-pepper” effect. Although this local error did not alter the overall ranking of the evaluated models, it indicates that the current pixel-based classification approach remains limited in its ability to exploit spatial contextual information in complex ecological transition zones (Hirayama et al., 2019; Lopatin et al., 2019). In addition, the dataset used in this study was acquired from a single study area during a single growing season. Therefore, the model’s generalization capability across different regions, years, and phenological stages remains unverified and requires further evaluation. A further limitation concerns the spatial validation design. Training and validation samples were separated at the ROI level, and no individual ROI was divided between the two subsets. However, no predefined geographic separation distance or spatial blocking scheme was imposed. A *post hoc* spatial check showed a minimum boundary distance of 0 m between some training and validation ROIs. Spatial autocorrelation may therefore persist among nearby samples, and the reported accuracy should be interpreted as an ROI-level holdout assessment rather than a fully spatially independent evaluation. In addition, the ROIs used for GLCM texture extraction varied in size (24 × 22 to 130 × 120 pixels), and each ROI was summarized as a single texture-descriptor vector rather than evaluated using a fixed-size moving window. Because GLCM statistics can be sensitive to the spatial scale of the analysis region, this variable ROI size may introduce scale-related differences in the extracted texture features across samples.

To address these limitations, three directions merit further investigation. First, to reduce the influence of mixed pixels and mitigate local salt-and-pepper effects, advanced classification approaches such as Object-Based Image Analysis (OBIA) and Convolutional Neural Networks (CNNs) could be incorporated. By leveraging spatial contextual information, these methods may enhance feature representation and improve classification stability in complex environments (Dewali et al., 2023; Ameslek et al., 2024). For the texture-extraction procedure, future studies could also evaluate the sensitivity of texture descriptors to ROI size or adopt standardized spatial windows to improve the consistency of texture representation. Second, to alleviate class confusion caused by spectral similarity in shrub–grass mixed communities, UAV-based LiDAR data or Canopy Height Models (CHMs) derived from oblique photogrammetry could be integrated to incorporate vertical structural information, thereby improving discrimination between shrub and herbaceous vegetation (Tarsha Kurdi et al., 2023; Fareed et al., 2026). Third, to further improve model robustness and transferability, future studies should incorporate radiometrically calibrated RGB data and multi-regional, multi-temporal observations covering key phenological stages, such as green-up and senescence periods, and adopt geographically blocked validation using independently separated ROIs. Such datasets and validation designs would help improve the physical interpretability of RGB-derived features and enhance the model’s spatiotemporal adaptability to complex grassland vegetation classification tasks.

## 5 Conclusions

This study evaluated UAV RGB and multispectral feature fusion, staged feature selection, and three classifiers for fine-scale mapping of four grassland land-cover types. The main findings are as follows:

1. Multi-source fusion combined spectral, vegetation-index, and texture information and improved discrimination among spectrally similar classes. Vegetation indices provided strong class-level signals, while low-correlation texture features contributed complementary spatial information.
2. Fusion order interacted with the feature selection algorithm rather than producing a universally superior strategy. Under the conditions evaluated in this study, Ts-SPA+LR achieved the highest observed validation accuracy (OA = 91.54%, Kappa = 88.72%) using only 10 features. However, its 0.63-percentage-point improvement over Direct-LR was not statistically significant, indicating that the main advantage of Ts-SPA+LR was the substantial reduction in feature dimensionality while maintaining comparable classification performance.
3. Under the conditions evaluated in this study, the proposed Ts-SPA+LR workflow provided a compact feature representation for fine-scale UAV-based grassland land-cover classification. Its robustness and applicability across different regions, years, and phenological stages remain to be validated before broader operational use.

## Data Availability

The raw data supporting the conclusions of this article will be made available by the authors, without undue reservation.
