# Supplementary material for: Fine-scale grassland land-cover classification using UAV RGB and multispectral imagery with staged feature selection
Source: Front Plant Sci. 2026 Oct 6;17:1956870. doi: 10.3389/fpls.2026.1956870 (PMC13680488; doi:10.3389/fpls.2026.1956870)
Supplement: Supplementary file 1 [file Table1.docx]

Supplementary Material

# 1 Supplementary Data

Table A1. Kruskal-Wallis statistics for the top 10 RF-SHAP features in the RGB and multispectral datasets.

| **Feature** | **Ac(Mean±Std)** | **As(Mean±Std)** | **Bl(Mean±Std)** | **Cm(Mean±Std)** | **H-value** | **P-value** |
| --- | --- | --- | --- | --- | --- | --- |
| NGBDI | -0.0141 ± 0.0091 | 0.0243 ± 0.0178 | -0.0466 ± 0.0134 | 0.0670 ± 0.0291 | 2263.25 | <0.001 |
| ENDI | 0.0082 ± 0.0376 | -0.1479 ± 0.0588 | 0.1213 ± 0.0404 | -0.2473 ± 0.0592 | 2259.43 | <0.001 |
| NDVI | 0.4603 ± 0.0474 | 0.6186 ± 0.0507 | 0.2530 ± 0.0709 | 0.6992 ± 0.0561 | 2251.85 | <0.001 |
| GBRI | 0.9404 ± 0.0209 | 1.0095 ± 0.0518 | 0.8797 ± 0.0265 | 1.1230 ± 0.0694 | 2244.17 | <0.001 |
| ExB | 0.1507 ± 0.0074 | 0.1282 ± 0.0163 | 0.1779 ± 0.0124 | 0.0839 ± 0.0257 | 2215.41 | <0.001 |
| NDCI | 0.3277 ± 0.0453 | 0.5212 ± 0.0517 | 0.1636 ± 0.0539 | 0.5736 ± 0.0572 | 2200.99 | <0.001 |
| OSAVI | 0.2819 ± 0.0317 | 0.4230 ± 0.0427 | 0.1843 ± 0.0465 | 0.4822 ± 0.0503 | 2194.69 | <0.001 |
| CIVE | 11.5028 ± 2.6025 | -9.4501 ± 5.6757 | 16.4744 ± 2.4869 | -0.9795 ± 5.5669 | 2193.7 | <0.001 |
| RDVI | 0.2324 ± 0.0275 | 0.3684 ± 0.0444 | 0.1674 ± 0.0396 | 0.4228 ± 0.0521 | 2141.6 | <0.001 |
| RGBRI | 0.0922 ± 0.0327 | 0.3327 ± 0.0661 | 0.0347 ± 0.0233 | 0.2667 ± 0.0743 | 2136.14 | <0.001 |
| VDVI | 0.0422 ± 0.0152 | 0.1489 ± 0.0301 | 0.0129 ± 0.0113 | 0.1311 ± 0.0374 | 2112.43 | <0.001 |
| ExG | 0.0574 ± 0.0210 | 0.2107 ± 0.0448 | 0.0174 ± 0.0154 | 0.1850 ± 0.0554 | 2111.29 | <0.001 |
| EVI2 | 0.2184 ± 0.0300 | 0.3815 ± 0.0559 | 0.1669 ± 0.0410 | 0.4460 ± 0.0682 | 2088.65 | <0.001 |
| TVI | 7.0518 ± 1.1684 | 13.9013± 2.5388 | 5.5678 ± 1.7930 | 16.4159±3.0029 | 2021.5 | <0.001 |
| LCI | 0.2042 ± 0.0238 | 0.2218 ± 0.0366 | 0.1113 ± 0.0351 | 0.3205 ± 0.0488 | 2003.39 | <0.001 |
| VARI | 0.2529 ± 0.0704 | 0.8804 ± 0.1994 | 0.2049 ± 0.0510 | 0.4487 ± 0.1465 | 1967.25 | <0.001 |
| R_MS | 15.7054 ± 2.0510 | 14.0557 ± 2.4039 | 41.3910 ± 9.5663 | 11.8420 ± 2.2459 | 1844.56 | <0.001 |
| B_RGB | 121.1426±12.6798 | 122.0527±16.2611 | 178.1368±23.3057 | 98.6364±16.4507 | 1695.81 | <0.001 |
| MDD | 0.7560 ± 0.0882 | 0.5963 ± 0.1267 | 0.5200 ± 0.1273 | 0.9693 ± 0.1872 | 1681.2 | <0.001 |
| IKAW | -0.1202 ± 0.0234 | -0.2920 ± 0.0489 | -0.1272 ± 0.0217 | -0.1428 ± 0.0536 | 1445.16 | <0.001 |

Note: Ac(Agropyron cristatum), As(Artemisia scoparia), Bl(bare land), Cm(Caragana microphylla).

**Table A2.** Normalized SHAP importance values of RGB and multispectral features.

| Rank | RGB Feature | SHAP(%) | Multispectral Feature | SHAP(%) |
| --- | --- | --- | --- | --- |
| 1 | NGBDI | 11.14 | R_MS | 10.16 |
| 2 | ExB | 10.38 | RDVI | 8.31 |
| 3 | GBRI | 8.90 | NDCI | 7.84 |
| 4 | IKAW | 8.80 | MDD | 7.51 |
| 5 | VDVI | 7.81 | EVI2 | 6.83 |
| 6 | ExG | 7.56 | ENDI | 5.53 |
| 7 | B_RGB | 6.36 | TVI | 5.48 |
| 8 | CIVE | 6.32 | NDVI | 5.44 |
| 9 | RGBRI | 5.34 | OSAVI | 5.40 |
| 10 | VARI | 3.95 | LCI | 5.00 |
| 11 | savg_rgb | 3.25 | NDRE | 4.92 |
| 12 | ExR | 2.58 | NDREI | 4.91 |
| 13 | MGRVI | 2.49 | NGI | 4.61 |
| 14 | G_RGB | 2.47 | G_MS | 4.54 |
| 15 | RGRI | 2.35 | NIR | 3.39 |
| 16 | entr_rgb | 2.11 | RE | 3.18 |
| 17 | R_RGB | 2.11 | GNDVI | 1.61 |
| 18 | MExG | 2.07 | entr_ms | 1.36 |
| 19 | cshade_rgb | 1.70 | TDVI | 1.34 |
| 20 | harcor_rgb | 1.69 | cshade_ms | 0.72 |
| 21 | NDI | 0.48 | harcor_ms | 0.62 |
| 22 | cont_rgb | 0.13 | savg_ms | 0.61 |
| 23 |  |  | TCARI | 0.41 |
| 24 |  |  | cont_ms | 0.28 |

Note: The sum of SHAP values may not be exactly 100% due to rounding errors.

Table A3. Vegetation indices extracted from UAV RGB and multispectral imagery.

| **Category** | **Index** | **Formula** |
| --- | --- | --- |
| Visible-light SIs | Excess Green Index (ExG) | $(2\times G-R -B)/(G+R+B)$ |
|  | Excess Blue Index (ExB) | $(1.4\times B-G)/(G+R+B)$ |
|  | Excess Red Index (ExR) | $(1.4\times R-G)/(G+R+B)$ |
|  | Modified Excess Green Index (MExG) | $1.262\times G-0.844\times R-0.311\times B$ |
|  | Visible-band Difference Vegetation Index (VDVI) | $(2\times G-R-B)/(2\times G+R+B)$ |
|  | Color Index of Vegetation Extraction (CIVE) | $0.441\times R-0.811\times G+0.385\times B+18.78745$ |
|  | Red-Green Ratio Index (RGRI) | $R/G$ |
|  | Visible Atmospherically Resistant Index (VARI) | $(G-R)/(G+R-B)$ |
|  | Modified Green Red Vegetation Index (MGRVI) | $(G^2-R^2)/( G^2+R^2)$ |
|  | RGB Redness Index (RGBRI) | $(G^2-R\times B)/( G^2+R\times B)$ |
|  | Green-Blue Ratio Index (GBRI) | $G/B$ |
|  | IKAW Index (IKAW) | $(R-B)/(R+B)$ |
|  | Normalized Green-Blue Difference Index (NGBDI) | $(G-B)/(G+B)$ |
|  | Normalized Difference Index (NDI) | $(G-R)/(G+R)$ |
| Multispectral SIs | Normalized Difference Vegetation Index (NDVI) | $(NIR-R)/(NIR+R)$ |
|  | Renormalized Difference Vegetation Index (RDVI) | $(NIR-R)/sqrt(NIR+R)$ |
|  | Normalized Difference Red-Edge Index (NDRE) | $(NIR-RE)/(NIR+RE)$ |
|  | Normalized Difference Chlorophyll Index (NDCI) | $(RE-R)/(RE+R)$ |
|  | Green Normalized Difference Vegetation Index (GNDVI) | $(NIR-G)/(NIR+G)$ |
|  | Multispectral Difference Descriptor (MDD) | $(NIR-RE)/(RE-G)$ |
|  | Two-band Enhanced Vegetation Index (EVI2) | $2.5\times(NIR-R)/(NIR+2.4\times R+1)$ |
|  | Normalized Green Index (NGI) | $G/(NIR+G+RE)$ |
|  | Optimized Soil-Adjusted Vegetation Index (OSAVI) | $1.16\times(NIR-R)/(NIR+R+0.16)$ |
|  | Transformed Vegetation Index (TVI) | $60\times(NIR-G)-100\times(R-G)$ |
|  | Enhanced Normalized Difference Index (ENDI) | $(R-G)/sqrt ((R^2+G^2)$ |
|  | Transformed Difference Vegetation Index (TDVI) | $sqrt(0.5+(NIR-R)/(NIR+R))$ |
|  | Transformed Chlorophyll Absorption Ratio Index (TCARI) | $3\times((RE-R)-0.2\times(RE-G) \times(RE/R))$ |
|  | Normalized Difference Red-Edge Index (NDREI) | $(RE-G)/(RE+G)$ |
|  | Leaf Chlorophyll Index (LCI) | $(NIR-RE)/(NIR+R)$ |

Note: SI: Spectral Index; For visible-band indices, R, G, and B denote R_RGB, G_RGB, and B_RGB, respectively; for multispectral indices, NIR, G, R, and RE denote NIR, G_MS, R_MS, and RE, respectively.

**Table A4. Sensitivity of feature-selection thresholds based on training-partition five-fold cross-validation.**

| Method | Threshold | Retained candidate features | Fixed k | 5-fold CV OA(%) | 5-fold CV Kappa (%) |
| --- | --- | --- | --- | --- | --- |
| SPA | 0.005 | 21 | 10 | 99.10 | 98.80 |
| SPA | 0.006 | 20 | 10 | 99.10 | 98.80 |
| SPA | **0.007** | **20** | 10 | 99.10 | 98.80 |
| SPA | 0.008 | 20 | 10 | 99.10 | 98.80 |
| SPA | 0.009 | 20 | 10 | 99.10 | 98.80 |
| mRMR | 0.085 | 26 | 10 | 98.87 | 98.49 |
| mRMR | 0.095 | 24 | 10 | 98.87 | 98.49 |
| mRMR | **0.105** | **22** | 10 | 98.87 | 98.49 |
| mRMR | 0.115 | 19 | 10 | 98.87 | 98.49 |
| mRMR | 0.125 | 15 | 10 | 98.87 | 98.49 |
| ReliefF | 0.07 | 31 | 6 | 97.34 | 96.46 |
| ReliefF | 0.08 | 29 | 6 | 97.34 | 96.46 |
| ReliefF | **0.09** | **25** | 6 | 97.85 | 97.14 |
| ReliefF | 0.10 | 17 | 6 | 98.44 | 97.92 |
| ReliefF | 0.11 | 15 | 6 | 98.36 | 97.81 |

**Note:** Candidate feature counts were obtained using the corresponding threshold on the training partition. For each threshold setting, the feature number (k) was fixed at the value used in the corresponding main analysis, and classification performance was evaluated using five-fold cross-validation within the training partition only. Logistic regression was used as the representative classifier. The selected thresholds are shown in bold.

**Table A5. Sensitivity of LR-based classification performance to random data partitioning.**

| Seed | Direct-LR OA(%) | Os-SPA-LR OA(%) | Ts-SPA-LR OA(%) | Ts-mRMR-LR OA(%) | Ts-ReliefF-LR OA(%) |
| --- | --- | --- | --- | --- | --- |
| 12 | 90.91 | 89.52 | 91.41 | 88.76 | 88.38 |
| 28 | 90.91 | 90.15 | 91.54 | 88.51 | 87.63 |
| 42 | 90.91 | 89.52 | 91.29 | 88.76 | 87.63 |
| 225 | 90.91 | 89.52 | 91.54 | 88.38 | 87.63 |
| 3047 | 91.92 | 90.66 | 91.29 | 88.38 | 87.63 |

**Note:** For each random seed, the train–validation partition was regenerated and the complete modeling procedure was repeated using the same feature-selection thresholds and hyperparameter search grids described in the main text. Feature selection and hyperparameter optimization were performed using the corresponding training data only. Differences in validation accuracy across seeds reflect variation in the data partition and the resulting cross-validation-selected models.
